# Beyond Direct Two-Electron Oxidation: High-Potential Formaldehyde Oxidation on Palladium Still Generates Hydrogen Atoms

**DOI:** 10.1021/jacs.6c14579

**Published:** 2026-09-23

**Authors:** Ruizhi Li, Yufei Li, Xiaosong Hu, Tao Gao, Yujie Sun

**Affiliations:** † Department of Chemistry, 2514 University of Cincinnati, Cincinnati, Ohio 45221, United States; ‡ Institute of Atomic and Molecular Physics, 12530 Sichuan University, Chengdu, Sichuan 610065, China

## Abstract

Electrochemical oxidation is typically regarded as incompatible with hydrogen intermediate formation, particularly at highly positive potentials where surface hydrogen species are expected to be rapidly oxidized. Formaldehyde oxidation reaction (FOR) on palladium represents a prominent example: low-potential FOR has been associated with hydrogen generation, whereas high-potential FOR is commonly interpreted as a direct two-electron oxidation to formate that bypasses hydrogen intermediates. Here we challenge this conventional view using a Janus palladium membrane electrode (J-Pd_m_) that spatially decouples hydrogen generation, absorption, permeation, and detection. By monitoring the hydrogen oxidation reaction (HOR) current on the electrochemically isolated detection side of J-Pd_m_, we directly quantify hydrogen atoms generated during FOR under alkaline conditions. Unexpectedly, permeated hydrogen remains detectable over a broad FOR potential window up to 1.5 V vs RHE (reversible hydrogen electrode), demonstrating that high-potential FOR on Pd is not a hydrogen-free oxidation process. Coulometric measurements confirm hydrogen uptake within the Pd lattice, with H/Pd ratios reaching up to 0.57. Spectroscopic and computational results support a sequential hydrogen-generating oxidation mechanism in which formaldehyde-derived intermediates undergo favorable C–H activation, while the resulting hydrogen atoms compete among absorption into the Pd lattice, recombination to H_2_, and oxidation to protons. FOR-generated hydrogen atoms can be utilized for styrene hydrogenation in an organic phase, establishing anodic hydrogenation as a viable electrosynthetic strategy. These findings provide new mechanistic understanding of formaldehyde oxidation on palladium and also demonstrate J-Pd_m_ as a powerful platform for detecting and utilizing transient hydrogen intermediates in anodic reactions.

## Graphical abstract

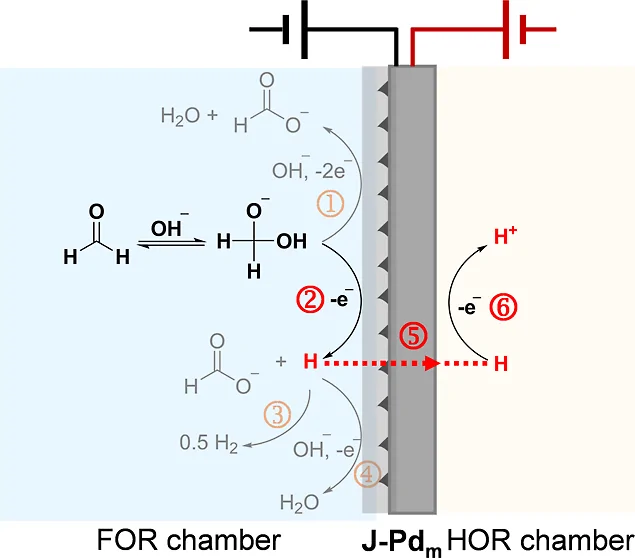

## Introduction

Hydrogen (H_2_) is a central chemical feedstock and a promising green energy carrier because it can store renewable energy and release energy without CO_2_ emission.^1^ At present, however, most industrial H_2_ is still produced from fossil resources, motivating the development of more sustainable routes driven by renewable energy.^2^ Electrocatalytic water splitting offers an appealing pathway for green H_2_ production under mild conditions, yet its broad implementation remains limited by the large voltage input required for the overall reaction.^3–5^ A major source of this inefficiency is the anodic O_2_ evolution reaction (OER), which is kinetically sluggish and produces O_2_ as a low-value product.^6–10^ To address this limitation, extensive efforts have been devoted to replacing OER with thermodynamically more favorable oxidation reactions, such as the oxidative valorization of biomass-derived molecules,^11–14^ alcohols,^15–17^ aldehydes,^18,19^ amines,^20–22^ urea,^23–25^ and other small molecules.^26^ Nevertheless, in most cases H_2_ is produced only at the cathode, while the anodic process primarily serves as an energy-saving or value-generating counter reaction. From the perspective of maximizing hydrogen output and electron economy, a more attractive strategy would be to produce H_2_ from both electrodes while simultaneously realizing a useful anodic oxidation reaction. Formaldehyde oxidation reaction (FOR) is particularly appealing in this context. In alkaline media, formaldehyde can be partially oxidized to formate, a value-added C_1_ product, while releasing hydrogen equivalents at an exceptionally low thermodynamic potential (Figure 1a). Prior works have shown that coupling FOR with HER can enable dual H_2_ production from both the anode and cathode at much lower voltage than conventional water electrolysis.^27–29^

**Figure 1:**
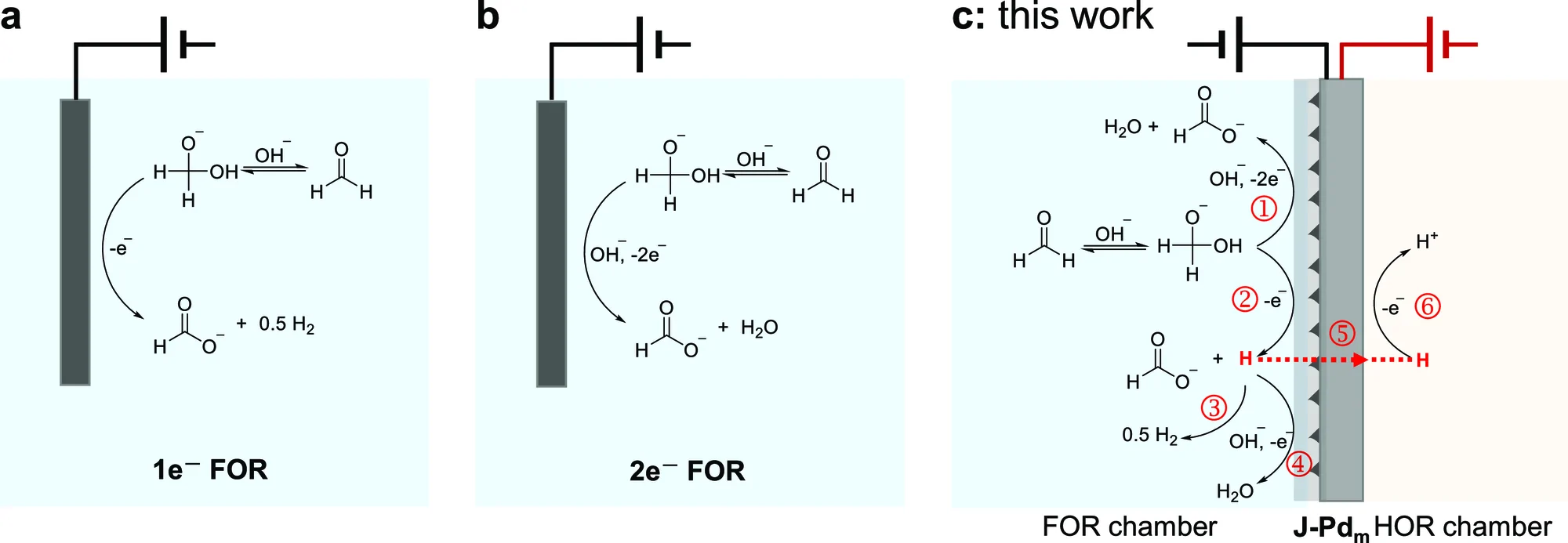
(a) One- and (b) two-electron oxidation pathways of FOR under alkaline conditions. (c) Schematic illustration of the J-Pd_m_ platform for electrochemical detection and quantification of permeated FOR-derived hydrogen.

These advances highlight FOR as an attractive anodic alternative to OER and suggest that formaldehyde may serve not only as an oxidizable substrate but also as a source of reactive hydrogen species. Palladium membrane electrodes offer a unique opportunity to interrogate and utilize such hydrogen species. Owing to the exceptional ability of Pd to absorb and transport atomic hydrogen, hydrogen atoms generated on one side of a Pd membrane can enter the Pd lattice, diffuse through the metal, and emerge on the opposite side.^30^ In our recent work, we exploited this property to demonstrate electrocatalytic dual hydrogenation, in which hydrogen atoms generated from low-potential FOR on a Pd membrane anode permeated through the membrane and drove organic hydrogenation in an electrochemically isolated chemical chamber.^31^

Despite this promise, a fundamental mechanistic question remains unresolved: does FOR on Pd actually generate hydrogen atoms under anodic conditions, especially at highly positive potential? Many studies of alkaline formaldehyde oxidation proposed that methylene glycolate dehydrogenation produces adsorbed hydrogen atoms, which can either recombine to form H_2_ (Figure 1a) or undergo electrochemical oxidation to protons.^32–36^ The classical works of van den Meerakker^37^ and Bindra^38^ concluded that on Group VIII metals such as Pt and Pd, formaldehyde oxidation proceeds without simultaneous H_2_ evolution because adsorbed hydrogen is rapidly oxidized. This conclusion has strongly influenced the conventional view that Pd, as an excellent hydrogen oxidation reaction (HOR) electrocatalyst, should not permit detectable hydrogen generation during anodic FOR. Moreover, high-potential FOR has often been interpreted as a direct two-electron oxidation process to formate that bypasses hydrogen intermediate formation altogether (Figure 1b).^39^ This interpretation is largely based on the absence of gaseous H_2_ as a product, rather than on direct detection of transient hydrogen atoms. As a result, it has remained unclear whether hydrogen atoms are truly absent at high anodic potentials during FOR (one direct 2e^–^-oxidation step) or whether they are formed but consumed too rapidly through HOR (two consecutive 1e^–^-oxidation steps).

Herein, we address this long-standing ambiguity using a Janus palladium membrane electrode (J-Pd_m_) specifically designed to decouple hydrogen generation, hydrogen absorption, hydrogen permeation, and hydrogen oxidation. As shown in (Figure 1c), in this architecture, FOR occurs on one side of J-Pd_m_ under alkaline anodic conditions, while the opposite side of J-Pd_m_ is electrochemically isolated and configured to detect permeated hydrogen atoms through HOR current. Because only hydrogen atoms absorbed into and transported through J-Pd_m_ can contribute to the HOR signal on the detection side, this platform provides an unambiguous electrochemical readout of hydrogen atom generation during FOR. Unlike conventional gas-collection measurements, which can miss hydrogen atoms that are rapidly oxidized at the FOR interface, our J-Pd_m_ platform directly probes the competing pathway in which hydrogen atoms enter the Pd lattice before interfacial oxidation. Using this design, we demonstrate that formaldehyde oxidation on Pd indeed generates hydrogen atoms under alkaline conditions, even at fairly positive anodic potential up to 1.5 V vs RHE (reversible hydrogen electrode). These results show that high-potential FOR on Pd is not simply a hydrogen-free, direct 2e^–^-oxidation process. Instead, hydrogen-generating elementary steps persist over a broad anodic potential range of FOR, while the fate of the generated hydrogen atoms is governed by competition among (i) recombination to H_2_, (ii) electrochemical oxidation on Pd (i.e., HOR), and (iii) absorption/permeation through the Pd membrane. Remarkably, hydrogen absorption into Pd can remain kinetically competitive with HOR even though Pd is widely recognized as an efficient HOR electrocatalyst. By providing direct, quantitative electrochemical evidence for permeated hydrogen atoms generated from anodic FOR, together with theoretical computation, this work establishes J-Pd_m_ as a powerful platform for resolving transient hydrogen intermediates in oxidation reactions and opens new opportunities for coupling value-added anodic oxidation with hydrogen utilization chemistry.

## Results and Discussion

Control electrochemical studies were first conducted to identify suitable conditions for subsequent J-Pd_m_ experiments. Formaldehyde oxidation was negligible in either acidic, or neutral electrolytes, or on bare glassy carbon (Figure S1), whereas Pd in alkaline electrolyte showed pronounced FOR activity, confirming that both Pd and alkaline conditions are required. Increasing KOH concentration enhanced FOR current (Figure S2), but high alkalinity also accelerated spontaneous Cannizzaro disproportionation of formaldehyde (Figures S3–S8). Therefore, 1 M KOH was selected to balance catalytic activity and substrate stability. The HCHO concentration was then optimized in 1 M KOH (Figure S9), where increasing HCHO from 1 mM to 0.5 M improved the oxidation current, while 1.0 M HCHO led to decreased activity and more severe background formate formation (Figures S10–S13). Based on these combined studies, 0.5 M HCHO in 1 M KOH was selected as the standard FOR electrolyte for subsequent J-Pd_m_ studies.

With the optimized FOR conditions established, we next constructed the J-Pd_m_ platform illustrated in Figure 1c to directly probe hydrogen intermediates generated during anodic formaldehyde oxidation. In this architecture, a thin Pd membrane electrode (0.025 mm) simultaneously serves as both the working electrode and the hydrogen-selective transport medium separating two electrochemically isolated compartments. The HCHO-containing side functions as the FOR chamber, where formaldehyde oxidation can proceed through competing pathways, including direct two-electron oxidation to formate (step ① in Figure 1c), hydrogen-generating one-electron oxidation (step ②), hydrogen recombination to H_2_ (step ③), local electrochemical oxidation of surface-adsorbed H* (step ④), and hydrogen absorption into and permeation through J-Pd_m_ (step ⑤). The opposite side of J-Pd_m_ serves as the HOR chamber, where permeated hydrogen atoms are oxidized under independently controlled anodic polarization (step ⑥). Therefore, any anodic current detected in this HOR chamber must originate from hydrogen atoms generated from formaldehyde oxidation in the FOR chamber. To improve FOR activity while maintaining efficient hydrogen transport, the FOR-facing side of J-Pd_m_ was electrodeposited with Pd nanoparticles. As compared in Figure S14, moderate Pd deposition enhanced FOR current, whereas excessive deposition decreased activity, likely due to reduced mass transport and/or impeded hydrogen accessibility through the membrane. Accordingly, a deposition charge of 1 C from Pd^2+^ precursor was adopted for all the J-Pd_m_ electrodes.

We then optimized the operating potential in the HOR chamber to ensure efficient oxidation of hydrogen atoms permeating from the FOR chamber. For these experiments, the FOR chamber was operated galvanostatically at constant anodic current densities of 5, 10, 20, 30, and 40 mA cm^–2^, while the HOR chamber contained 0.5 M H_2_SO_4_ and the HOR-facing side of J-Pd_m_ was biased at different potentials from 0.5 to 1.5 V vs RHE with an interval of 0.2 V for 20 min. As shown in Figure 2a–e, the detected HOR current density generally increased as the applied HOR potential became more positive, confirming that higher anodic bias more effectively oxidizes permeated hydrogen species. At relatively low FOR current densities of 5 and 10 mA cm^–2^, the HOR responses at 1.1, 1.3, and 1.5 V vs RHE were comparable, suggesting that hydrogen oxidation was sufficiently fast under these conditions and that the measured current became primarily limited by the rate of hydrogen generation and permeation through the Pd membrane. For example, when 10 mA cm^–2^ was applied in the FOR chamber, the HOR current reached approximately 8 mA cm^–2^ at high HOR potentials, indicating that a large fraction of the FOR-generated hydrogen flux could be captured and oxidized on the detection side. In contrast, at higher FOR current densities of 20, 30, and 40 mA cm^–2^, the dependence of the HOR response on the applied detection potential became more pronounced. Under these larger hydrogen-flux conditions, lower HOR potentials were insufficient to fully oxidize the permeated hydrogen, whereas 1.5 V vs RHE consistently produced the highest HOR current density. This trend indicates that, as the rate of hydrogen generation and loading in the FOR chamber increases, a stronger anodic driving force is required in the HOR chamber to effectively oxidize those permeated hydrogen atoms.

**Figure 2:**
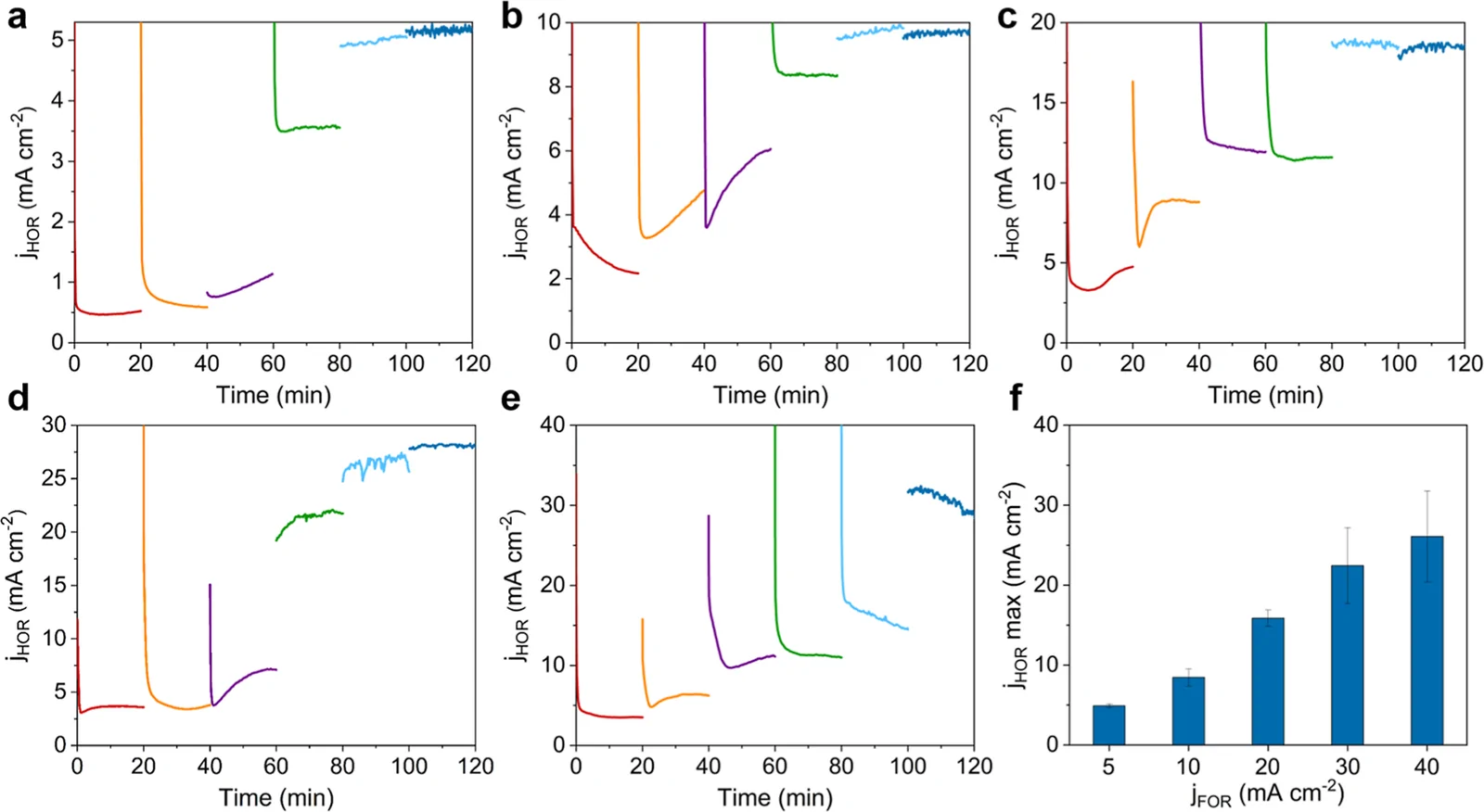
(a–e) Time-dependent HOR current density measured at applied potentials ranging from 0.5 to 1.5 V vs RHE with an interval of 0.2 V in the HOR chamber while the FOR current density was set at (a) 5, (b) 10, (c) 20, (d) 30, and (e) 40 mA cm^–2^ in the FOR chamber. The electrolyte in the HOR chamber was 0.5 M H_2_SO_4_, while the electrolyte in the FOR chamber was 1.0 M KOH with 0.5 M HCHO. (f) Comparison of the maximum HOR current densities detected at 1.5 V vs RHE upon the application of different FOR current densities. Data points are presented as mean values and error bars indicate standard deviations obtained from triplicate (*n* = 3) measurements.

The maximum HOR current densities obtained upon the application of different FOR current densities are summarized in Figure 2f. The HOR current increased monotonically with increasing applied FOR current density, reaching approximately 26 mA cm^–2^ when the FOR chamber was operated at 40 mA cm^–2^. Importantly, sustaining this FOR current requires a highly positive anodic potential of approximately 1.4 V vs RHE in the FOR chamber, as shown by the corresponding chronopotentiometric measurement in Figure S15. This observation indicates that FOR-derived hydrogen capable of permeating through the Pd membrane is still generated even at strongly anodic FOR potentials, which will be further discussed in the subsequent paragraphs.

To further validate the origin of the detected HOR signal, control experiments were performed under the selected FOR/HOR operating potentials (Figure S16). Negligible current was observed in either chamber in the absence of HCHO, whereas the addition of 0.5 M HCHO immediately initiated FOR and was also accompanied by the development of a substantial HOR current at the opposite Pd surface. This direct temporal correlation between HCHO addition, FOR, and the subsequent HOR response, together with the absence of measurable HOR current prior to HCHO addition, rules out an appreciable contribution from background electrochemical processes associated with the Pd membrane or pre-existing hydrogen stored within the membrane. These results therefore further confirm that the detected HOR current originates from hydrogen generated during FOR and subsequently transported through the Pd membrane.

Having established the appropriate HOR-side potential and verified the FOR-dependent origin of the detected HOR current using our J-Pd_m_ platform, we next employed this strategy to investigate how the generation and permeation of FOR-derived hydrogen intermediates evolve as a function of the applied FOR potential. Since OER becomes appreciable on Pd electrodes above ca. 1.6 V vs RHE in 1 M KOH (Figure S17), the FOR-side potential was systematically varied from 0.3 to 1.5 V vs RHE with an interval of 0.2 V. In these experiments, the FOR chamber was operated potentiostatically in 1.0 M KOH containing 0.5 M HCHO, while the opposite HOR chamber was held at 1.5 V vs RHE to promote efficient oxidation of hydrogen atoms permeating through the Pd membrane. To assess whether the detected hydrogen flux was affected by the electrolyte environment and HOR kinetics on the detection side, the HOR chamber was independently tested using 0.5 M H_2_SO_4_, 1.0 M KPi, and 1.0 M KOH as the electrolytes.

As shown in Figure 3a–c, increasing the applied FOR potential initially increased both the FOR current density and the corresponding HOR current density, indicating that the flux of FOR-derived hydrogen entering and permeating through the Pd membrane initially increases with the anodic driving force. In 0.5 M H_2_SO_4_, for example, the HOR current density increased from ca. 4 mA cm^–2^ at an FOR potential of 0.3 V vs RHE to a maximum of ca. 17 mA cm^–2^ when the FOR potential increased to 0.7 V vs RHE (Figure 3a). Upon further increasing the applied FOR potential, however, the detected HOR current gradually decreased. Importantly, measurable HOR currents were still observed at FOR potentials above 1.0 V, including ca. 8.8 mA cm^–2^ at 1.3 V and ca. 2 mA cm^–2^ at 1.5 V, indicating that FOR-derived hydrogen remains detectable even at highly positive anodic potentials.

**Figure 3:**
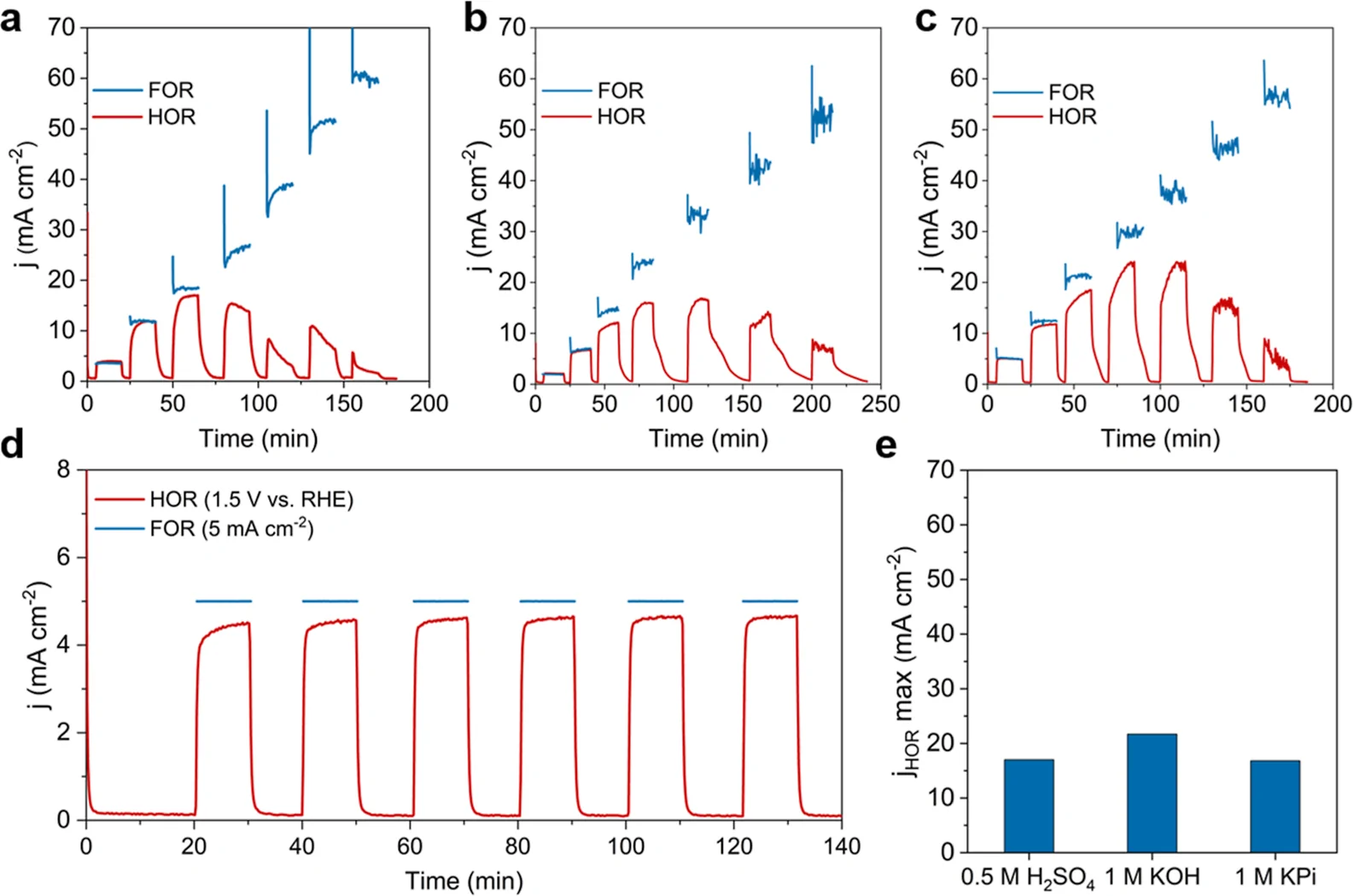
Time-dependent HOR current densities measured at 1.5 V vs RHE using (a) 0.5 M H_2_SO_4_, (b) 1 M KPi, and (c) 1 M KOH as the HOR electrolytes and 1.0 M KOH with 0.5 M HCHO as the FOR electrolytes when FOR was carried out at potentials varying from 0.3 to 1.5 V vs RHE with an interval of 0.2 V. (d) Correlated response between the FOR and HOR current densities during periodic FOR on/off operation. (e) Comparison of the maximum HOR current densities obtained in (a–c).

The dynamic coupling between FOR and hydrogen permeation was further examined by periodically switching FOR on and off (Figure 3d). Specifically, FOR was maintained for 10 min and then switched off for 10 min, with the on/off operation repeated for six consecutive cycles. Upon initiation of FOR, the HOR current developed at the opposite side of the Pd membrane, whereas interruption of FOR caused the HOR current to rapidly return to the baseline. This response was reproducibly recovered over multiple consecutive on/off cycles without an apparent progressive loss, demonstrating a reversible temporal correlation between FOR-derived hydrogen generation and its permeation through the Pd membrane. The structural stability of the Pd membrane under the reaction conditions was further evaluated by XRD (Figures S18 and S19) and SEM/EDS (Figures S20–S24) before and after electrolysis. The characteristic diffraction peaks of metallic Pd were retained after electrolysis without the appearance of a detectable crystalline palladium oxide phase, although minor changes in surface morphology and an increase in the oxygen content were observed. These results indicate that the Pd membrane largely retains its metallic crystalline structure, with only limited surface compositional and morphological changes during electrolysis. Together with the negligible background current observed in the absence of HCHO (Figure S16) and the reproducible FOR/HOR response (Figure 3d), these observations collectively indicate that sustained or irreversible oxidation of the Pd membrane does not make an appreciable contribution to the detected HOR current under the present operating conditions.

With these results in hand, we next considered the origin of the decreasing HOR response at higher FOR potentials. This decline suggests that, although hydrogen intermediates continue to be generated during FOR, a smaller fraction of the generated hydrogen is ultimately transported through the Pd membrane and detected at the HOR-facing interface. Several competing pathways may account for this decreased fraction of hydrogen detected by HOR. Specifically, on the FOR-facing side of J-Pd_m_, the generated H* can either enter the Pd lattice or recombine to form H_2_ before permeation. In addition, at increasingly positive FOR potentials, surface H* can undergo local direct electrochemical oxidation (H* + OH^–^ → H_2_O + e^–^). Therefore, not all hydrogen intermediates generated during FOR are necessarily transported through the Pd membrane and detected as HOR current. Consequently, the detected HOR current only represents the fraction of FOR-derived hydrogen that is successfully transported through the Pd membrane and electrochemically captured at the HOR-facing side, rather than the total amount of hydrogen intermediates generated during FOR. Accordingly, the decrease in HOR current at higher FOR potentials does not necessarily indicate the disappearance of hydrogen-intermediate formation but rather reflects increasing competition between hydrogen permeation and alternative hydrogen-consumption pathways.

Similar potential-dependent trends were obtained when neutral KPi or alkaline KOH was used in the HOR chamber (Figure 3b,c). In fact, the maximum HOR current densities showed only modest electrolyte-dependent variation (Figure 3e). These results demonstrate that high-potential FOR on Pd is not a purely hydrogen-free, direct two-electron oxidation process. Instead, hydrogen-generating steps persist across a broad anodic potential window (up to 1.5 V vs RHE), while the fate of the generated hydrogen progressively shifts from Pd-lattice absorption/permeation toward local electrochemical oxidation at the FOR interface.

Next, we sought to quantify how much of the hydrogen atoms generated from FOR could be stored within the Pd lattice under anodic polarization. The hydrogen amount in J-Pd_m_ was expressed as the H/Pd ratio and determined using a coulometric discharge approach.^40–44^ In these measurements, constant-current FOR electrolysis was conducted at 10, 20, 30, and 40 mA cm^–2^ until the Pd membrane reached a steady hydrogen-loaded state, as indicated by the plateaued oxidation current in the HOR chamber (Figure S25a). The FOR electrolysis was then stopped, and the HOR side was biased at 1.5 V vs RHE in 0.5 M H_2_SO_4_, a condition identified above to efficiently oxidize permeated hydrogen species. Under this anodic discharge condition, hydrogen stored within the Pd lattice was oxidized and depleted from J-Pd_m_ (Figure S25b), and the corresponding oxidation charge was integrated to determine the amount of absorbed hydrogen (Figure S25c). As summarized in Figure 4a, appreciable hydrogen loading was achieved in J-Pd_m_ under anodic FOR conditions. The calculated H/Pd ratios increased from 0.40 at an applied FOR current density of 10 mA cm^–2^ to 0.49, 0.50, and 0.51 at 20, 30, and 40 mA cm^–2^, respectively. This trend indicates that increasing the rate of FOR-driven hydrogen generation promotes hydrogen uptake into the Pd lattice, although the H/Pd ratio begins to level off at higher current densities. To further evaluate whether the discharge electrolyte influenced the measured hydrogen content, analogous measurements were performed using 1.0 M KPi and 1.0 M KOH as the electrolyte of the HOR chamber while maintaining the FOR current density at 40 mA cm^–2^. Comparable H/Pd ratios were obtained in these electrolytes as well, reaching 0.574 in 1.0 M KPi and 0.567 in 1.0 M KOH (Figure 4b). These results confirm that the substantial hydrogen loading stored in J-Pd_m_ is not limited to acidic HOR detection conditions but instead reflects genuine hydrogen absorption into the Pd lattice during FOR. To the best of our knowledge, it represents the first quantitative determination of hydrogen content in Pd generated under anodic polarization (i.e., FOR). It should be noted that the measured H/Pd ratios are lower than those typically obtained during cathodic hydrogen loading from water reduction on Pd, which is not surprising given the different hydrogen-generation environment. Under cathodic conditions, hydrogen is continuously supplied directly through water reduction at the Pd electrode surface. In contrast, under anodic FOR conditions, hydrogen formation/absorption must compete against its local electrochemical oxidation to protons at the J-Pd_m_ interface. Nevertheless, the ability to achieve H/Pd ratios beyond 0.57 under anodic FOR conditions demonstrates that a significant fraction of FOR-generated hydrogen can enter and accumulate within the Pd lattice, providing a quantitative basis for the hydrogen permeation and downstream hydrogenation chemistry described below.

**Figure 4:**
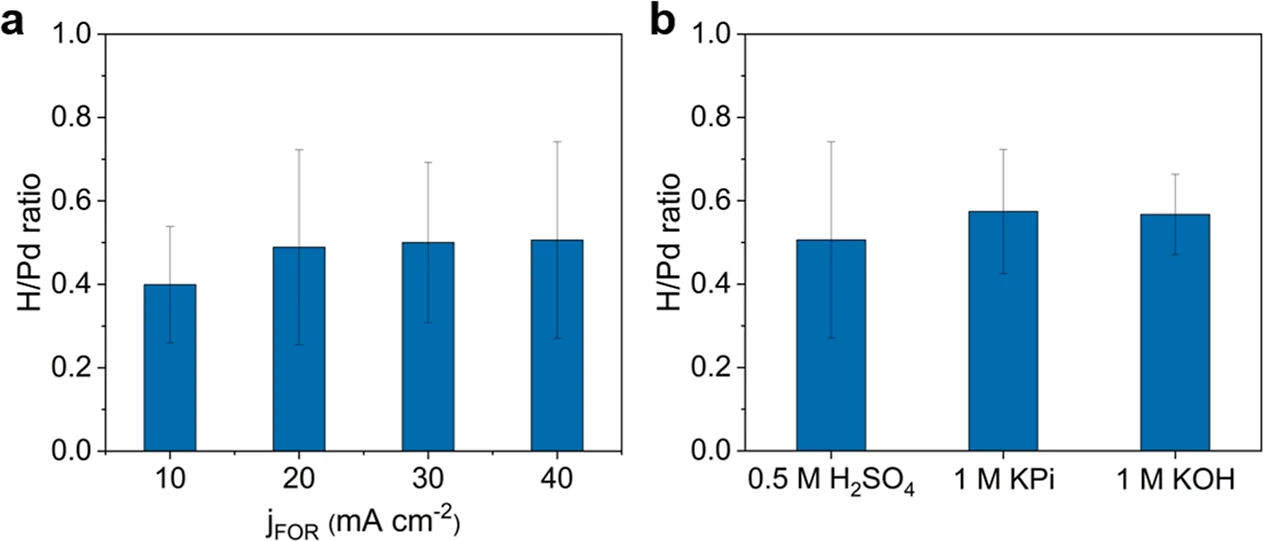
(a) Measured H/Pd ratio of J-Pd_m_ after 30 min electrolysis of FOR at different current densities when the HOR chamber was filled with 0.5 M H_2_SO_4_ and the FOR chamber was filled with 0.5 M HCHO in 1.0 M KOH. (b) Measured H/Pd ratio of J-Pd_m_ after 30 min electrolysis of FOR at 40 mA cm^–2^ when the HOR chamber was filled with different electrolytes. Data points are presented as mean values and error bars indicate standard deviations obtained from triplicate (*n* = 3) measurements.

To determine whether those hydrogen atoms generated from FOR could be directly utilized for synthetic hydrogenation in an organic phase, we selected styrene hydrogenation in methanol as a model reaction. Styrene was chosen because it is poorly soluble in water and therefore represents a typical hydrophobic organic substrate. In addition, its hydrogenation produces ethylbenzene as the sole hydrogenation product, allowing straightforward quantification of conversion and hence Faradaic efficiency (FE).^45,46^ Meanwhile, a deposition charge of 5 C from Pd^2+^ precursor was adopted for the Pd that faces the chemical chamber to enhance the substrate adsorption. As shown in Figure 5a, a three-compartment electrochemical palladium membrane reactor was employed for this study. The FOR chamber was charged with 1.0 M KOH and 0.5 M HCHO, while the chemical chamber contained 50 mM styrene in methanol. Constant-current FOR electrolysis was performed at current densities ranging from 5 to 40 mA cm^–2^ to drive hydrogen generation, permeation through the Pd membrane, and final utilization in styrene hydrogenation in the chemical chamber. Each reaction was terminated after passing the theoretical amount of charge required for complete styrene hydrogenation. As plotted in Figures 5b and S26, styrene hydrogenation proceeded successfully under all investigated FOR current densities, confirming that hydrogen generated during anodic formaldehyde oxidation remains chemically accessible for downstream hydrogenation in an organic phase. At an applied FOR current density of 5 mA cm^–2^, more than 43 mM ethylbenzene was produced, corresponding to a Faradaic efficiency (FE) of 86%. To further explore whether the permeated hydrogen atoms are directly responsible for styrene hydrogenation, an additional control experiment was performed by applying 0 V vs Fc^+/0^ (Fc: ferrocene) to the chemical chamber while maintaining FOR at 5 mA cm^–2^. The applied potential was calibrated against the Fc^+/0^ redox couple (Figure S27). Under these conditions, time-dependent product analysis revealed substantially suppressed styrene hydrogenation, with only ∼6% ethylbenzene formed after 8 h (Figure S28). Meanwhile, a sustained anodic current was recorded at the chemical-facing Pd surface throughout the 8 h electrolysis (Figure S29), indicating continuous electrochemical consumption of the permeated hydrogen under applied anodic polarization. We therefore attribute the suppressed hydrogenation to competition between electrochemical oxidation of the permeated hydrogen and its chemical utilization for styrene hydrogenation. In contrast, under open-circuit conditions, the permeated hydrogen remains available at the chemical-facing Pd surface for the hydrogenation of styrene to ethylbenzene.

**Figure 5:**
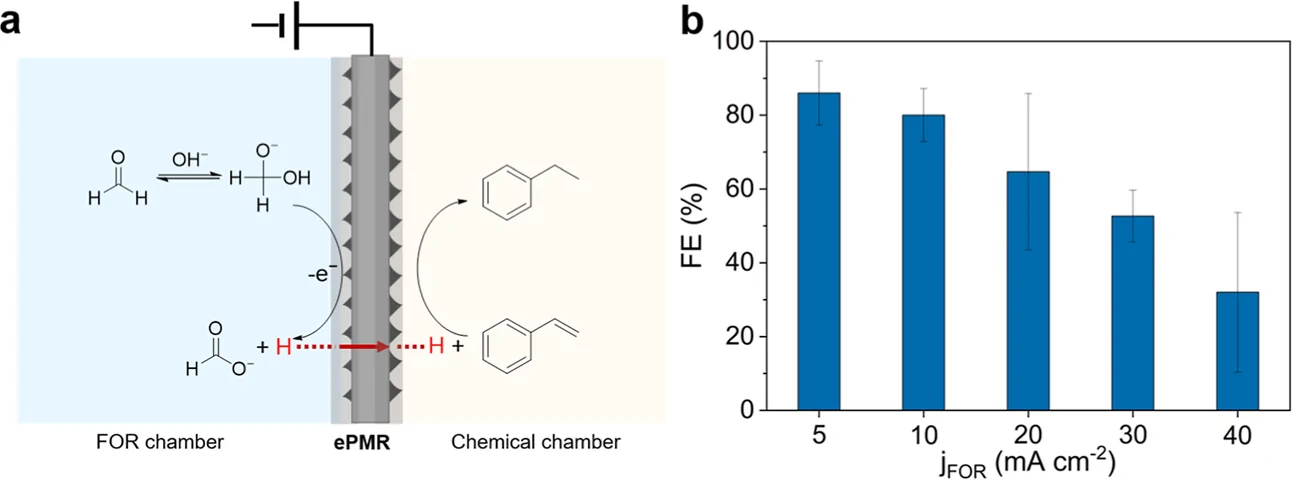
(a) Schematic illustration of styrene hydrogenation using permeated hydrogen from FOR. Chemical chamber: 50 mM styrene; FOR chamber: 0.5 M HCHO in 1.0 M KOH. (b) Faradaic efficiency (FE) of styrene hydrogenation to ethylbenzene at different FOR current densities after passing the theoretical amount of required charge assuming 100% FE. Data points are presented as mean values and error bars indicate standard deviations obtained from triplicate (*n* = 3) measurements.

As the applied FOR current density increased, both ethylbenzene production and FE gradually decreased. This trend is consistent with the mechanistic results described above: at higher anodic current densities and more positive FOR potentials, a larger fraction of the generated hydrogen intermediates is consumed by local electrochemical oxidation at the FOR interface before it can enter and permeate through the Pd lattice. Nevertheless, the observation of ethylbenzene formation even at elevated FOR current densities confirms that FOR-generated hydrogen can be transported across J-Pd_m_ and used for organic hydrogenation under anodic operating conditions.

To further probe the surface species formed during FOR and provide spectroscopic support for the hydrogen-involved oxidation pathway, potential-dependent in situ FTIR spectroscopy was performed under different conditions. Spectra were collected from open-circuit conditions to 1.5 V vs RHE (Figure 6). In 1.0 M KOH without HCHO, only the characteristic vibrational features associated with interfacial or adsorbed water were observed (Figure S30).^47,48^ In contrast, upon the addition of 0.5 M HCHO, two characteristic bands at ca. 1589 and 1346 cm^–1^ increased progressively with increasing potential (Figure 6a). These bands are assigned to the asymmetric and symmetric OCO stretching vibrations, respectively, of formate-related species, indicating the progressive formation of formate during FOR as the applied potential becomes more positive.^49–52^ Importantly, a weak feature near ca. 1904 cm^–1^ emerged at higher anodic potentials. This band is tentatively assigned to a Pd–H stretching vibration, suggesting the formation of a hydrogen-involved surface state during FOR.^53–56^ Although this assignment should be treated cautiously because of the weak intensity of the band, its appearance under anodic FOR conditions is consistent with the electrochemical detection of permeated hydrogen and the coulometric evidence for hydrogen loading in J-Pd_m_. To further examine this assignment, isotope-labeling experiments were performed using deuterated formaldehyde (Figure S31). With 0.5 M DCDO, the formate-related bands shifted to ca. 1572 and 1322 cm^–1^,^57^ respectively, while the weak feature at ca. 1904 cm^–1^ was no longer resolved as a distinct band. This behavior is consistent with the formation of Pd–D species upon H/D substitution, with the corresponding Pd–D stretching vibration expected in the ca. 1340 cm^–1^ region based on the reported isotope shifts of Pd–H/Pd–D vibrations.^58^ The resulting Pd–D vibration would therefore substantially overlap with the DCOO-related band at ca. 1322 cm^–1^, making it difficult to resolve as an independent feature. Direct comparison of the spectra collected at 0.7 V vs RHE further supports this interpretation, showing the loss of the distinct ca. 1904 cm^–1^ feature together with the isotope-induced shifts of the formate-related bands (Figure 6b). Together, these in situ FTIR results support the conclusion that high-potential FOR involves both formate formation and hydrogen-associated surface intermediates, rather than proceeding exclusively through a hydrogen-free direct two-electron oxidation pathway.

**Figure 6:**
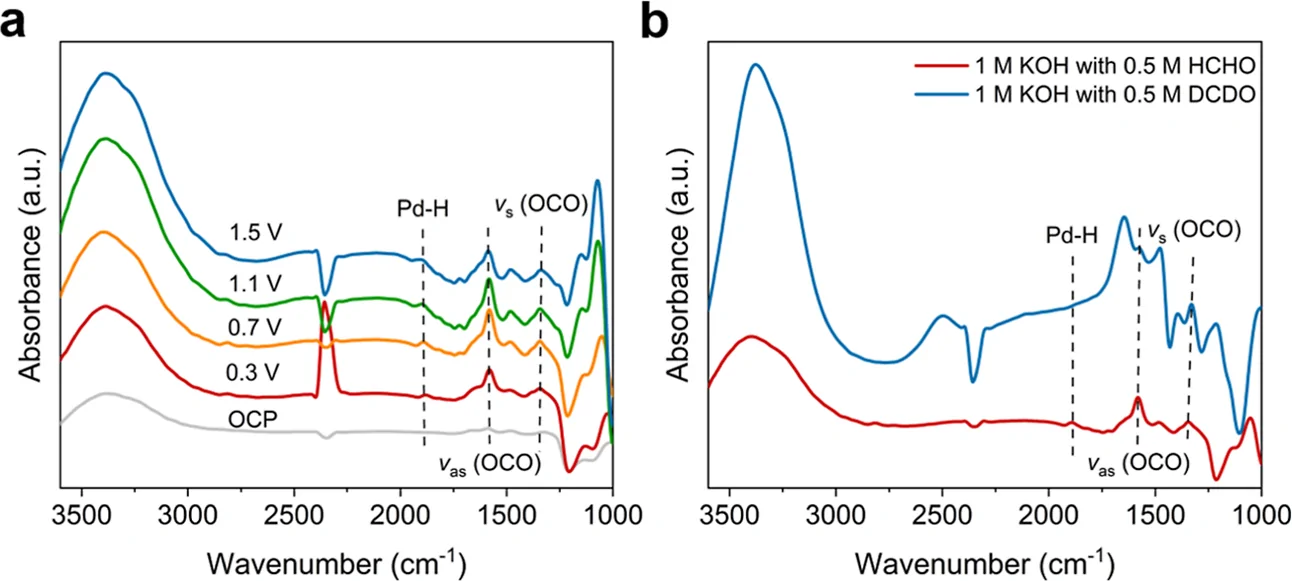
(a) Potential-dependent in situ ATR–FTIR spectra of electrocatalytic FOR on Pd in 1 M KOH with 0.5 M HCHO. (b) Comparison of the ATR–FTIR spectra measured in 1 M KOH, 1 M KOH with 0.5 M HCHO, and 1 M KOH with 0.5 M DCDO at 0.7 V vs RHE.

To further support the above experimental results, density functional theory (DFT) calculations were performed using Pd(111) and hydrogen-enriched PdH_0.6_(111) surfaces as model catalysts (Figure S32). Pd(111) was selected as the representative surface because the XRD pattern of the Pd membrane is dominated by the (111) reflection, and Pd(111) is also the most thermodynamically stable low-index facet of fcc Pd that has been widely used in theoretical studies of hydrogen adsorption and hydride formation on palladium.^59^ For the Pd(111) slab, the bottom two atomic layers were fixed to represent the bulk lattice, whereas the upper two layers were fully relaxed. To model hydrogen-enriched palladium under FOR conditions, a nonstoichiometric PdH_0.6_(111) surface was constructed from the Pd(111) slab. Because palladium hydrides are characterized by disordered hydrogen distributions, a combined random-loading and ab initio molecular dynamics (AIMD) approach was used and the detailed description of DFT computations can be found in the Supporting Information.

We first examined the energetics of C–H bond activation, the key hydrogen-generating elementary step during formaldehyde oxidation. Under neutral conditions, molecular formaldehyde (HCHO) and hydrated formaldehyde (HCH­(OH)_2_) were both considered. As presented in Figure 7a, HCHO exhibits an energy barrier of 0.642 eV for the homolytic cleavage of its C–H bond on Pd, while a much higher energy barrier (1.604 eV) was computed on PdH_0.6_. Figure 7b compares the C–H bond activation profiles of HCH­(OH)_2_ on Pd versus PdH_0.6_, both of which exhibit large energy requirements, 1.500 and 1.604 eV, respectively. These results indicate that it is less likely to observe hydrogen atom release from either HCHO or HCH­(OH)_2_ on Pd under neutral conditions, in agreement with the experimental results. In contrast, remarkably different computational results were obtained under alkaline conditions. As shown in Figure 7c, the deprotonated methylene glycolate intermediate HCH­(OH)­O^–^ was examined, because this species is expected to dominate in strongly basic media.^60^ The calculated free-energy profiles show that C–H bond cleavage is quite favorable for HCH­(OH)­O^–^ on Pd under alkaline conditions, with an activation barrier of 0.514 eV. In fact, the C–H bond activation of HCH­(OH)­O^–^ on PdH_0.6_ was calculated to be spontaneous without an observable barrier. These results indicate that the alkaline intermediate HCH­(OH)­O^–^ is intrinsically more susceptible to hydrogen-generating C–H activation. This trend is consistent with the experimental observation that formaldehyde oxidation on Pd is strongly promoted under alkaline conditions, whereas activity is negligible in neutral electrolyte.

**Figure 7:**
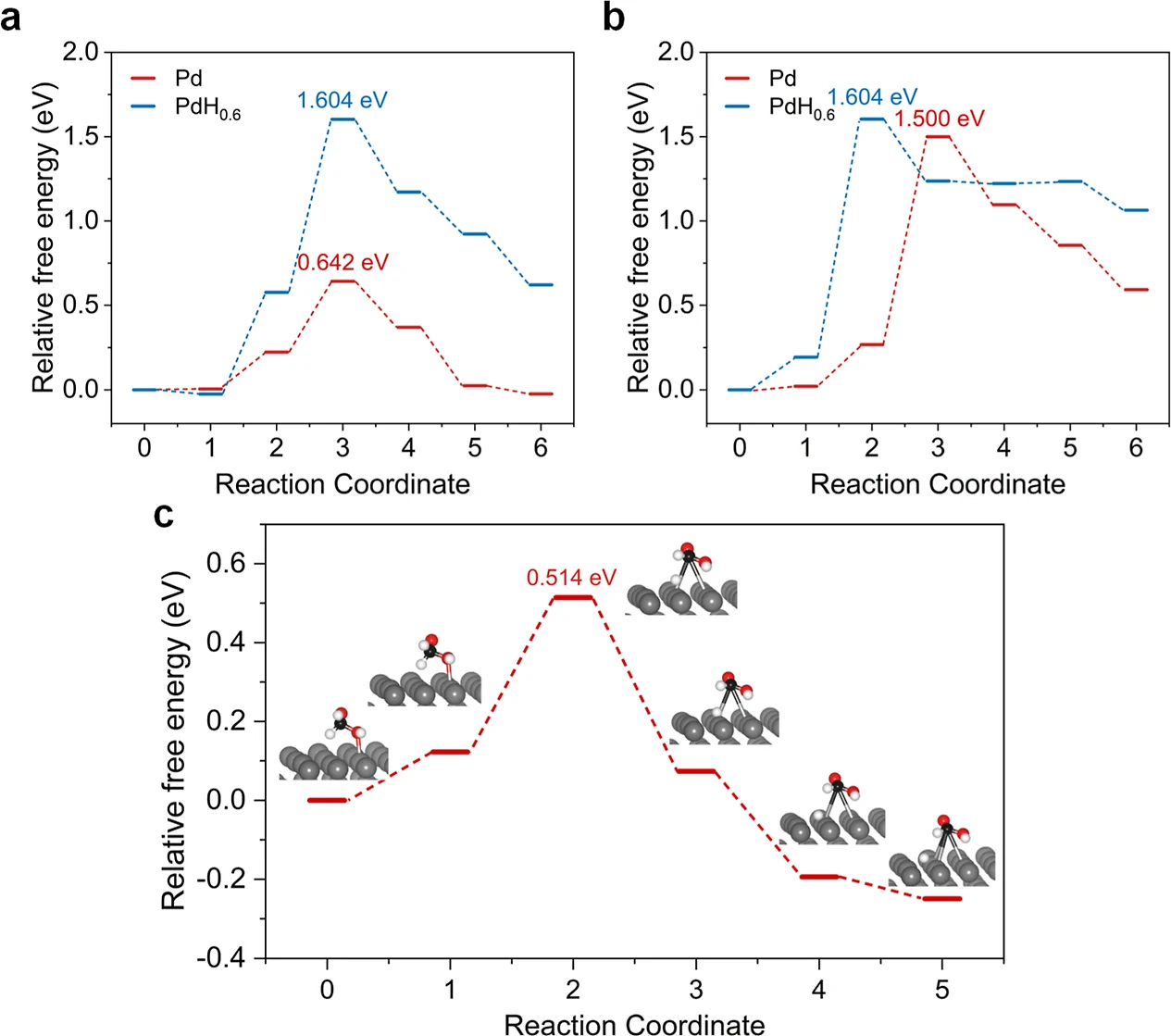
(a,b) Calculated free-energy profiles for C–H bond cleavage of HCHO (a) and HCH­(OH)_2_ (b) under neutral conditions on Pd(111) and PdH_0.6_(111). (c) Calculated free-energy profiles and the corresponding atomic configurations for C–H bond cleavage of HCH­(OH)­O^–^ under alkaline conditions on Pd(111).

We next examined the possible fate of hydrogen intermediates generated after the C–H bond cleavage of HCH­(OH)­O^–^ under alkaline conditions on Pd. Two kinetically competing pathways were considered: (i) absorption of surface hydrogen into the subsurface Pd lattice and (ii) recombination of surface hydrogen atoms to evolve molecular H_2_. As shown in Figure 8, hydrogen absorption into the subsurface region of Pd proceeds with a low activation barrier of 0.225 eV, whereas H_2_ formation requires a much higher barrier of 1.668 eV. These calculations indicate that once hydrogen atoms are generated during alkaline FOR, their absorption into Pd is kinetically more favorable than recombinative H_2_ evolution. This preference for subsurface hydrogen absorption provides a theoretical basis for the experimentally observed hydrogen permeation across J-Pd_m_ and the substantial hydrogen loading quantified within the Pd membrane under anodic FOR conditions (Figure 4).

**Figure 8:**
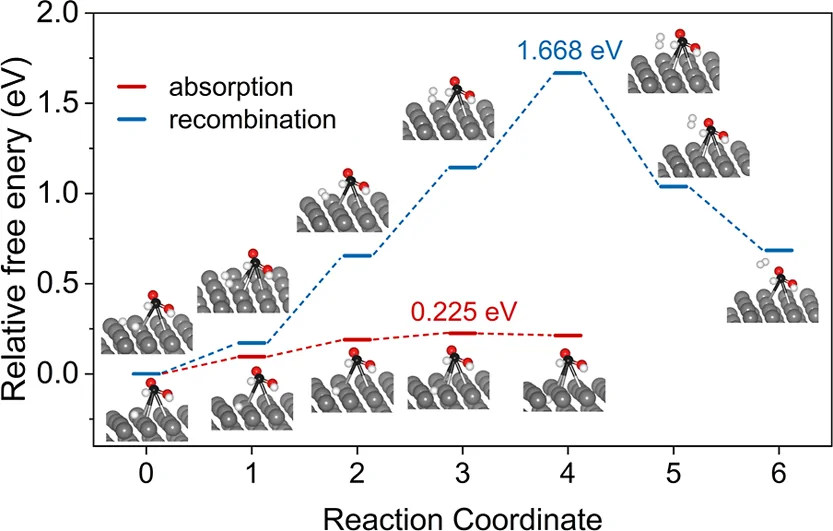
Calculated free-energy profiles and the corresponding atomic configurations for the absorption into Pd and recombination of hydrogen intermediates generated during FOR on Pd(111).

Because C–H bond activation of HCH­(OH)­O^–^ was calculated to proceed spontaneously on PdH_0.6_, we next evaluated whether the resulting carbon-containing intermediates could be stabilized on the hydrogen-enriched Pd surface. Figure S33 compares the adsorption strengths of several representative intermediates such as *HC^•^OHO^–^ (the barrierless C–H bond cleavage product of adsorbed HCH­(OH)­O^–^ on PdH_0.6_), *HCOOH, and *HCOO^–^ on PdH_0.6_. Among these species, *HC^•^OHO^–^ shows the strongest adsorption stability (−6.209 eV), indicating a particularly strong interaction between this intermediate and the hydrogen-rich Pd surface. This result suggests that although PdH_0.6_ can strongly promote the initial hydrogen-generating C–H activation step, the resulting strongly bound carbonaceous intermediates may accumulate on the surface in the absence of an external driving force. Such surface accumulation would limit sustained spontaneous formaldehyde conversion. Under electrochemical FOR conditions, however, anodic polarization can continuously oxidize these adsorbed intermediates toward more weakly bound products like HCOOH and HCOO^–^, thereby regenerating active Pd sites and maintaining catalytic turnover. This interpretation is consistent with the observation that spontaneous formaldehyde conversion is not sustained over extended reaction times (Figure S34), whereas electrochemical FOR proceeds continuously under applied potential.

To further clarify whether hydrogen-generating oxidation remains viable under anodic polarization, we investigated the potential-dependent energetics of HCH­(OH)­O^–^ oxidation on both Pd and PdH_0.6_ under alkaline conditions. In this proposed sequential pathway, HCH­(OH)­O^–^ first undergoes C–H bond cleavage to generate a surface hydrogen intermediate, followed by subsequent oxidation of the carbon-containing intermediate toward formate. As shown in Figure 9a, the C–H activation barrier on Pd decreases markedly with increasing applied potential. At *U* = 0 V, this step requires an activation barrier of 0.541 eV. When the potential is increased to 0.3 V vs RHE, the barrier decreases to 0.214 eV; while at potentials above 0.6 V vs RHE, C–H cleavage becomes nearly barrierless. This trend indicates that anodic polarization not only promotes formaldehyde oxidation thermodynamically but also facilitates the initial hydrogen-generating C–H activation step kinetically. A similar potential-dependent analysis was then performed on the hydrogen-enriched PdH_0.6_ surface. In contrast to metallic Pd, C–H bond cleavage on PdH0.6 proceeds spontaneously without an observable activation barrier across the investigated potential range (Figure 9b), further confirming that hydrogen-rich Pd surfaces strongly facilitate hydrogen generation from HCH­(OH)­O^–^.

**Figure 9:**
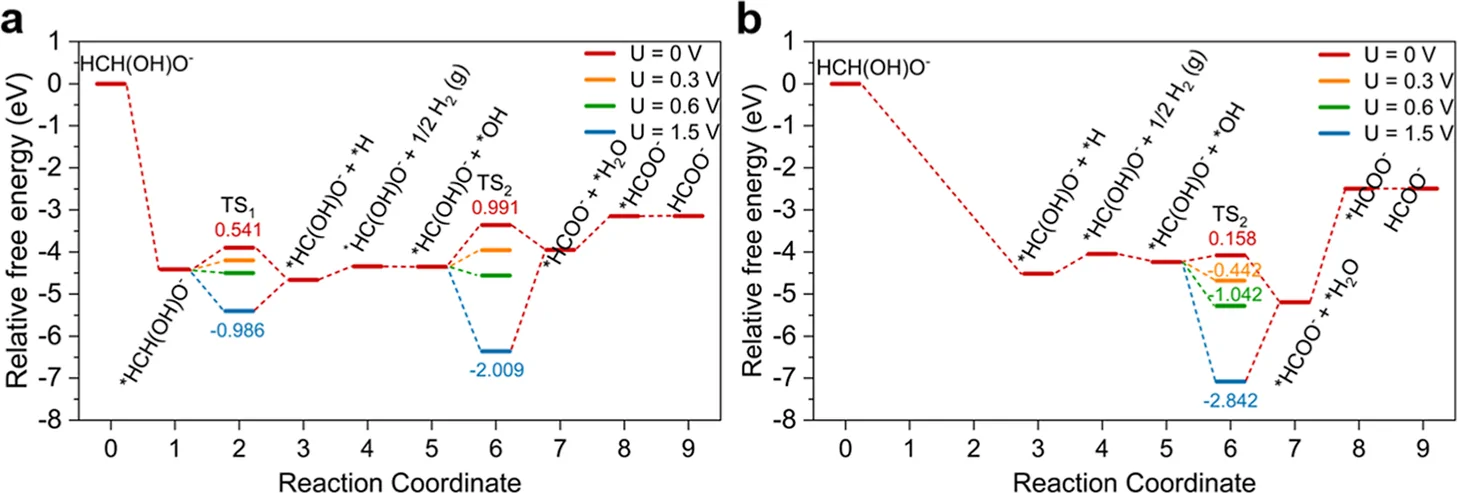
Potential-dependent free-energy profiles for the conversion of HOCH_2_O^–^ to HCOO^–^ and H_2_ on (a) Pd(111) and (b) PdH_0.6_(111) under alkaline conditions at the indicated electrode potentials (U = 0, 0.3, 0.6, and 1.5 V vs RHE).

We then examined the subsequent oxidation step following C–H activation. On Pd, this process is thermodynamically uphill at *U* = 0 V, with a calculated reaction free energy of 0.991 eV. Increasing the applied potential progressively stabilizes the oxidized products and lowers the energetic requirement associated with the second oxidation step. Consequently, the free-energy penalty for converting *HOCHO^–^ + *OH to *HCOO^–^ + H_2_O decreases markedly with increasing potential, indicating that the two-electron oxidation pathway becomes increasingly accessible under anodic conditions. The same potential-promoted trend is observed on PdH_0.6_, where the reaction free energy decreases from 0.158 eV at *U* = 0 V to −0.442 eV at *U* = 0.3 V vs RHE and becomes increasingly favorable at higher anodic potentials. Together, these calculations demonstrate that anodic polarization facilitates both C–H activation and subsequent oxidation in the sequential FOR pathway. Importantly, the hydrogen-generating pathway remains energetically accessible over the full potential window examined.

## Conclusions

In summary, this work reveals that formaldehyde oxidation on palladium remains a hydrogen-generating process even under highly anodic conditions where it has conventionally been regarded as a direct, hydrogen-free two-electron oxidation pathway. By using a Janus palladium membrane electrode that spatially decouples formaldehyde oxidation from hydrogen detection, we directly quantified permeated hydrogen atoms generated during FOR over a broad potential window up to 1.5 V vs RHE. We demonstrate that high-potential FOR proceeds through hydrogen-involved sequential oxidation pathways, while the fate of the generated hydrogen atoms is governed by competition between absorption into J-Pd_m_ and electrochemical oxidation on the FOR side of J-Pd_m_. Our theoretical calculations further show that alkaline HCH­(OH)­O^–^ undergoes favorable C–H activation on Pd and that hydrogen absorption into subsurface Pd is kinetically preferred over hydrogen recombination to yield H_2_. Potential-dependent free-energy profiles further demonstrate that anodic polarization promotes the sequential oxidation pathway without suppressing the initial hydrogen-generating step. Beyond resolving the mechanistic ambiguity of FOR, this study establishes J-Pd_m_ as a powerful platform for probing short-lived hydrogen intermediates during anodic oxidation reactions. More broadly, the ability to generate, transport, quantify, and utilize anodically produced hydrogen atoms opens new opportunities for coupling aldehyde oxidative transformations with downstream hydrogenation chemistry.

## Supplementary Material



## Notes

Experimental and computational details; Pd membrane preparation and characterization; electrochemical measurements; hydrogen permeation and coulometric experiments; operando ATR–FTIR spectra; DFT calculations; and Figures S1–S34 (PDF)
