# Supplementary material for: Beyond Direct Two-Electron Oxidation: High-Potential Formaldehyde Oxidation on Palladium Still Generates Hydrogen Atoms
Source: J Am Chem Soc. 2026 Sep 23;148(39):42583–93. doi: 10.1021/jacs.6c14579 (PMC13651521; doi:10.1021/jacs.6c14579)
Supplement: Supplementary file 1 [file ja6c14579_si_001.pdf]

Supporting Information

Beyond Direct Two-Electron Oxidation: High-Potential Formaldehyde Oxidation on Palladium Still Generates Hydrogen Atoms

Ruizhi Li,[1,+] Yufei Li,[2,+] Xiaosong Hu,[1,+] Tao Gao[2,*], and Yujie Sun[1,*]

[1]Department of Chemistry, University of Cincinnati, Cincinnati, Ohio 45221, United States

[2]Institute of Atomic and Molecular Physics, Sichuan University, Chengdu, Sichuan 610065, China

[+]These authors contributed equally to this work

[*]Corresponding author: gaotao@scu.edu.cn; yujie.sun@uc.edu

Experimental section

Materials

All chemicals were used as received without any further purification. Palladium foil (99.9%, 25 μm thickness) was purchased from Thermo Fisher Scientific. Anion exchange membrane (Fumasep FAA-3-50) was purchased from FuelCellStore. Deionized (DI) water with the resistivity of 18.2 MΩ·cm was obtained by the Barnstead E-Pure system. Palladium chloride (≥99.9%), potassium hydroxide (≥85%), formaldehyde (37 wt.% in H_2O), methanol (≥99.9%), styrene (>99.9%), formaldehyde-d_2 (20 wt.% in D_2O) were purchased from Sigma-Aldrich. Potassium phosphate monobasic anhydrous (ACS grade) was purchased from Amresco. Potassium phosphate dibasic anhydrous (99.16%) was purchased from Chem-impex Int'l Inc. Tertiary-Butanol was purchased from Fisher Scientific. Hydrochloric acid (37 wt.% in H_2O) was purchased from Carolina. Sulfuric acid (95-97%) was purchased from Supelco. Potassium chloride (≥99%) and 1,3,5-trimethoxybenzene (98%) was purchased from Ambeed.

Catalyst preparation

Electrodeposition was employed to prepare palladium nanoparticle-modified palladium membrane electrodes (J-Pdm). Commercial palladium membranes were first rinsed thoroughly with deionized water and mounted in an H-type electrochemical cell as the working electrode, thereby separating the two chambers. The exposed geometric area of the Pd membrane was 0.2 cm^2. An aqueous electrodeposition solution containing 15.9 mM $PdCl_2$ and 1.0 M HCl was introduced into one compartment, while a carbon rod and a saturated Ag/AgCl electrode were used as the counter and reference electrodes, respectively. Palladium electrodeposition was carried out potentiostatically at −0.2 V vs. Ag/AgCl until a total charge of either 1 or 5 C had been passed.

When a noticeable decline in membrane activity was observed, the Pd membrane was regenerated by sequential washing with HCl and deionized water, followed by thermal annealing at 850 °C for 3 h under a flowing 5% H_2/Ar atmosphere to restore the crystalline Pd lattice. After annealing, the membrane was cooled to room temperature and stored in air prior to reuse. Prior to each electrocatalytic experiment, the Pd membranes were thoroughly cleaned using a concentrated $HNO_3/H_2O/H_2O_2$ mixture (1:2:1, v/v/v) for 30 min until vigorous gas evolution subsided, followed by rinsing with ethanol and DI water and drying under N_2.

Electrochemical measurements

All electrochemical measurements were performed using a VMP-3 potentiostat (Biologic Science Instruments). Linear sweep voltammetry (LSV) measurements were conducted using a rotating disk electrode (RDE) system equipped with a modulated-speed rotator (PINE Research Instrumentation) at room temperature in a conventional three-electrode configuration. Pd-modified glassy carbon electrodes or bare glassy carbon electrodes (0.196 cm^2) were used as the working electrodes, while a carbon rod served as the counter electrode. Hg/HgO was used as the reference electrode in alkaline electrolytes, whereas Ag/AgCl was employed in KPi and H_2SO_4 electrolytes. Unless otherwise specified, LSV curves were collected under Ar atmosphere at a scan rate of 5 mV/s and a rotation speed of 1500 rpm without iR compensation. Commercial formaldehyde solution (37 wt%) was used directly as the oxidation substrate without further purification.

For the Janus palladium membrane (J-Pdm) configuration, a palladium membrane was mounted in an H-type electrochemical cell as the working electrode, thereby separating the reactor into two

compartments, denoted as the formaldehyde oxidation (FOR) chamber and hydrogen oxidation (HOR) chamber, respectively. The membrane surface facing the FOR chamber was modified by electrodeposition of 1 C of Pd nanoparticles. In both chambers, an anion exchange membrane (AEM) was used to separate the anodic and cathodic compartments. In the FOR chamber, 1 M KOH was used as the electrolyte on both sides of the AEM, and 0.5 M HCHO was introduced into the anodic compartment. A three-electrode configuration was employed, with the Pd membrane serving directly as the working electrode, Hg/HgO as the reference electrode, and a carbon rod as the counter electrode. In the HOR chamber, a separate three-electrode configuration was used, in which the same Pd membrane again served as the working electrode and a carbon rod was used as the counter electrode. Hg/HgO was employed as the reference electrode in 1 M KOH electrolyte, whereas Ag/AgCl was used in 1 M KPi and 0.5 M H_2SO_4 electrolytes. All potentials measured herein were converted to the reversible hydrogen electrode (RHE) scale through calibration using a Pt foil electrode in H_2-saturated electrolyte.

Physical characterization

The morphological features of the samples were characterized by scanning electron microscopy (SEM, FEI XL30) operated at 15 kV. X-ray diffraction (XRD) patterns were recorded using a Philips X'Pert Pro PW3040/00 (PANalytical) diffractometer with Cu Kα radiation (λ = 1.5406 Å), operated at 45 kV and 40 mA. Scans were collected over a 2θ range of 30° to 90°.

Hydrogenation of styrene

Electrochemical hydrogenation of styrene was conducted using an electrochemical palladium membrane reactor (ePMR). A palladium membrane was mounted in an H-type cell as the working electrode, thereby separating the reactor into two compartments, denoted as the FOR chamber and the chemical chamber. The membrane surface facing the FOR chamber was modified by electrodeposition of 1 C of Pd nanoparticles, whereas the opposite surface facing the chemical chamber was electrodeposited with 5 C of Pd nanoparticles. The chemical chamber contained 3 mL of MeOH solution with 50 mM styrene as the hydrogenation substrate. The FOR chamber contained 1 M KOH with 0.5 M HCHO, where constant-current electrolysis was performed to drive hydrogen generation and permeation through the Pd membrane. Electrolysis was terminated after passing a total charge of 29 C, corresponding to the theoretical charge required for the reaction. The reaction mixture was subsequently stirred for an additional 2 h prior to product quantification.

Product analysis

All reactants and products were identified and quantified by ^{1}H nuclear magnetic resonance (^{1}H NMR) spectroscopy using a Bruker AVANCE 500 MHz spectrometer.

For quantification of formate, 100 μL of the electrolyte was mixed with 500 μL of an internal standard solution containing 10 mM *tert*-butanol in water (1 mM *tert*-butanol in water was used if only 0.1 M HCHO was added). Spectra were collected using a water suppression method.

For quantification of styrene hydrogenation products, 100 μL of the reaction solution was mixed with 500 μL of an internal standard solution containing 10 mM 1,3,5-trimethoxybenzene in methanol. Spectra were likewise acquired using a water suppression method. Product quantification and styrene conversion were determined by peak integration analysis. Resonances

at 7.00–7.70 ppm corresponded to the aromatic protons of both styrene and ethylbenzene. The resonance at approximately 6.2 ppm was assigned to an internal standard 1,3,5-trimethoxybenzene. The resonance at approximately 5.8 ppm was assigned to the vinyl proton of styrene and used for quantitative analysis of styrene consumption, while the triplet at 1.20 ppm was assigned to the methyl group of ethylbenzene.

In situ ATR-FTIR

In situ attenuated total reflection Fourier transform infrared (ATR-FTIR) spectroscopy was performed using a Bruker INVENIO FT-IR spectrometer equipped with a liquid nitrogen-cooled LN-MCT detector at a resolution of 4 cm^{-1} and 64 scans per spectrum. A dual-layer metal structure consisting of gold and palladium was sequentially assembled on a crystalline silicon ATR prism. This configuration enables efficient infrared reflection at the Au/Pd interface. During measurements, a Hg/HgO reference electrode and a carbon rod counter electrode were used. The electrolyte consisted of 1.0 M KOH with 0.5 M HCHO or DCDO. Before data acquisition, the spectrometer was purged with Ar for 30 minutes to remove atmospheric CO_2 and moisture. Electrochemical control of the potential and electrolysis time was maintained using a CHI 760E potentiostat.

Computational details

The first principles calculations based on DFT were performed implementing Vienna Ab initio Simulation Package. (1) Core electrons were described by the projector augmented wave (PAW) potentials, (2) where valence electrons of Pd, H, O, C and K are considered with $4d^9 5s^1$, $1s^1$, $2s^2 2p^4$, $2s^2 2p^2$, and $3s^2 3p^6 4s^1$, respectively. Perdew-Burke-Ernzerhof exchange-correlation energy in the framework of generalized gradient approximation was adopted. (3) The kinetic energy cutoff of 400 eV was adopted for the plane wave expansion of the valence electron wave functions. Γ-centered Monkhorst-Pack k-points were sampled on a 1 × 1 × 1 grid for the structural relaxation of surface structures. The convergence criteria of 10^{-5} eV in energy and 0.05 eV $Å^{-1}$ in force were adopted during structural optimizations. Explicit models of the solution environment were considered in the calculations, and the final study model was derived from the stable structure after AIMD simulation. Ab initio molecular dynamics (AIMD) simulations were performed in the canonical (NVT) ensemble at 300 K, where the temperature was controlled using the Nosé-Hoover thermostat. The equations of motion were integrated with a time step of 1 fs. For the electronic structure calculations at each step, the energy convergence criterion for the self-consistent field (SCF) loop was set to 10^{-4} eV $cell^{-1}$. The climbing image nudged elastic band (CINEB) method was applied to search for the transition state and sample the energy profile of C-H bond fracture behavior. (4)

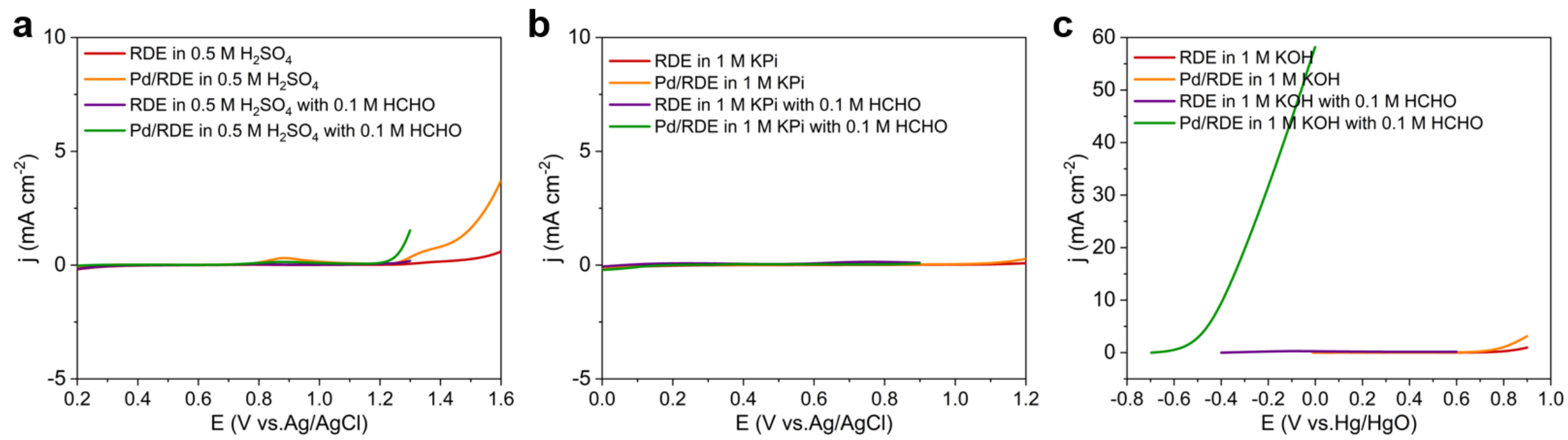

Figure S1. LSV curves recorded on RDE and Pd/RDE in Ar-saturated (a) 0.5 M H_2SO_4, (b) 1 M KPi, and (c) 1 M KOH with/without 0.1 M HCHO at 1500 rpm with a scan rate of 5 mV/s.

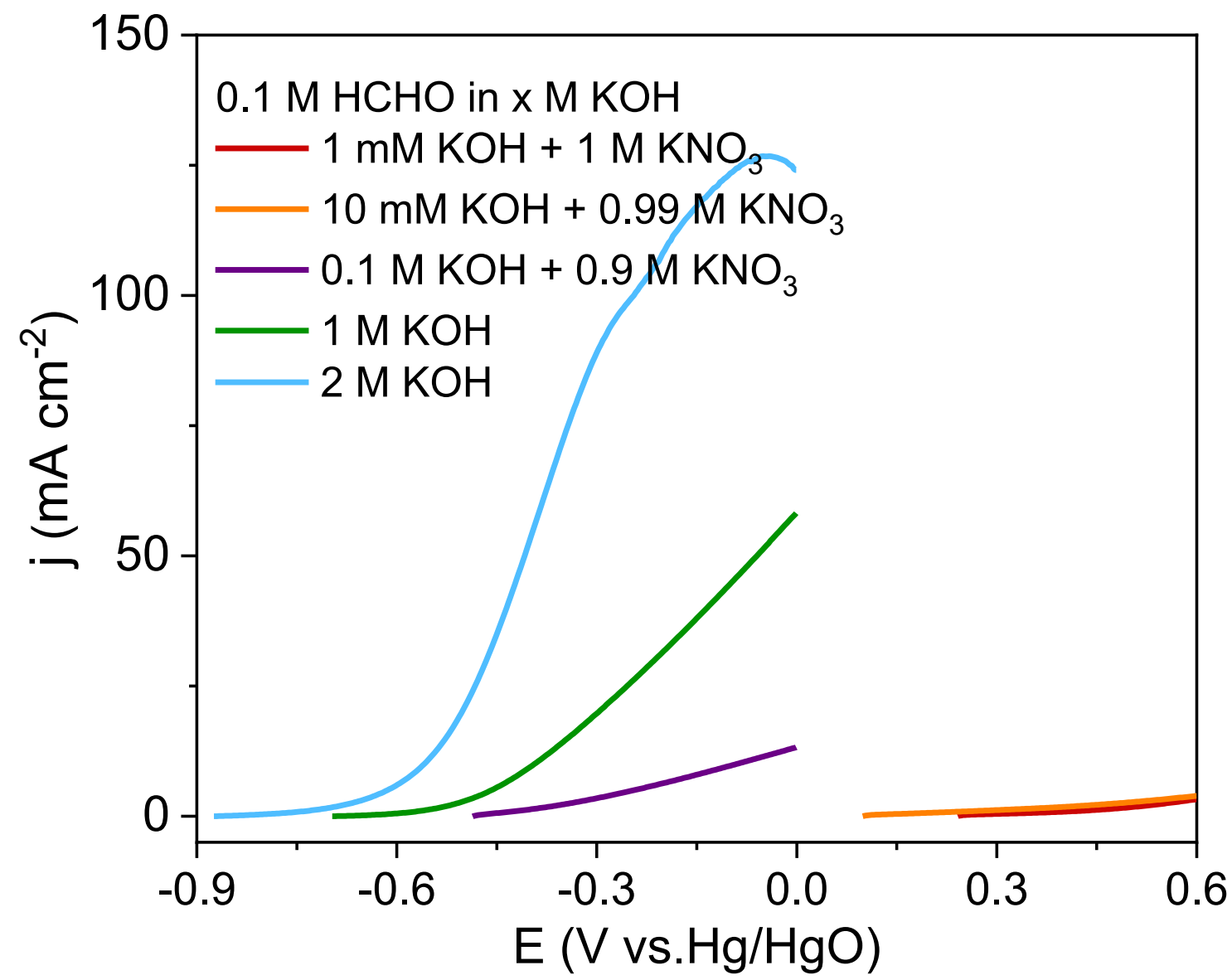

Figure S2. LSV curves recorded on Pd/RDE in Ar-saturated different concentrations of KOH with 0.1 M HCHO at 1500 rpm with a scan rate of 5 mV/s.

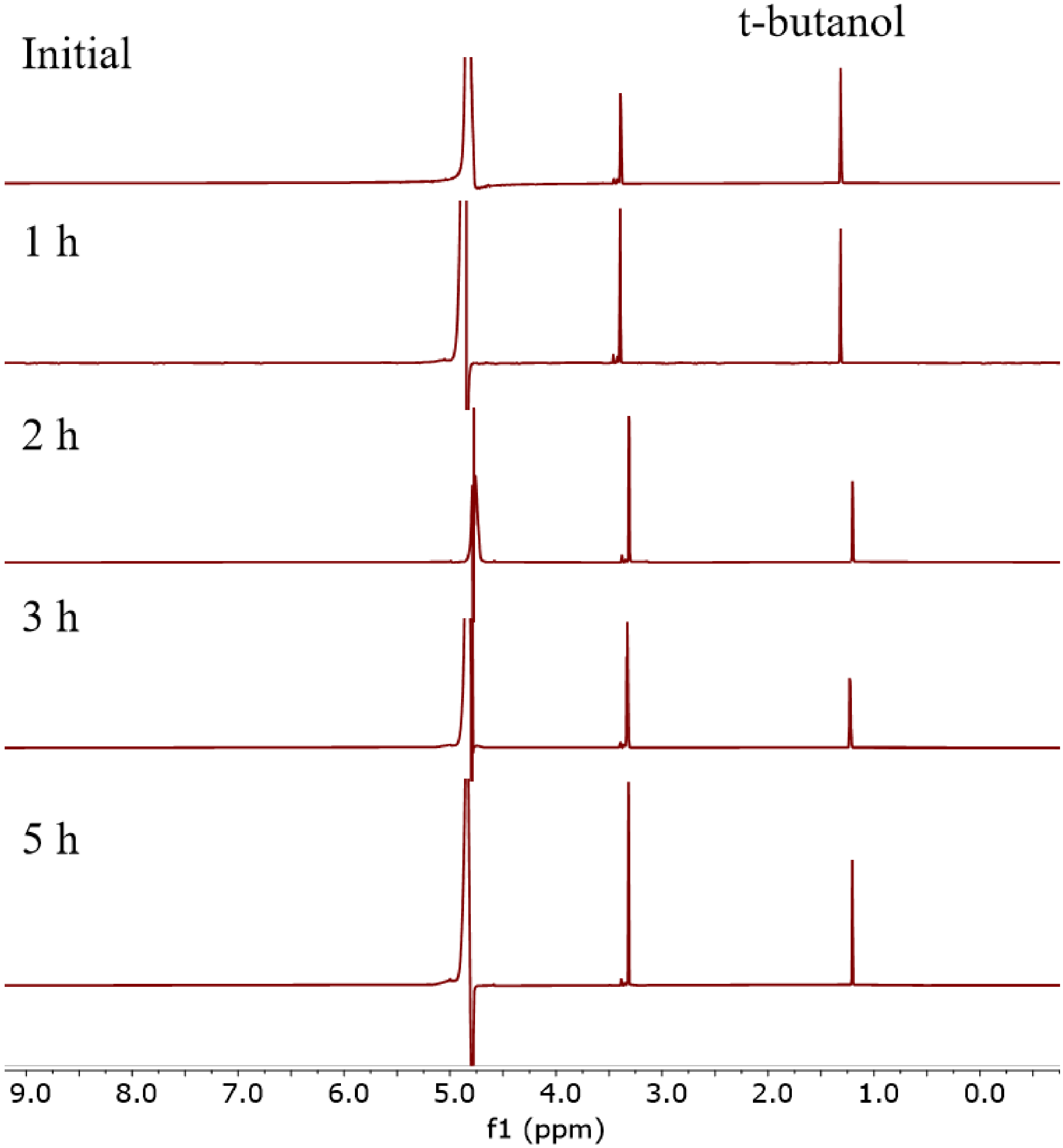

Figure S3. Time-dependent ^1H NMR spectra showing the Cannizzaro side reaction when dissolving 0.1 M HCHO in 1 mM KOH. 1 mM t-butanol was used as an internal standard and water suppression method was employed.

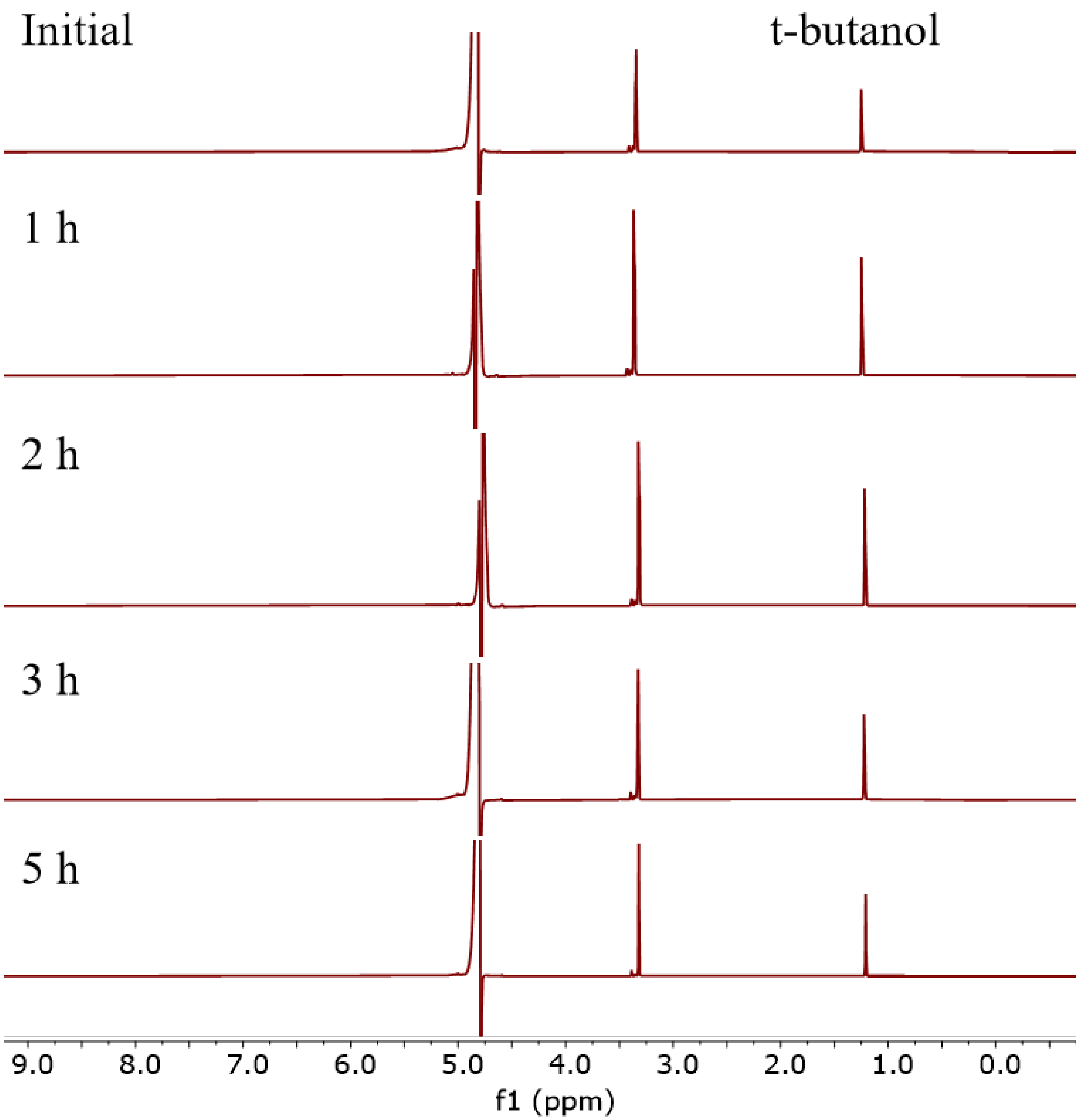

Figure S4. Time-dependent ^1H NMR spectra showing the Cannizzaro side reaction when dissolving 0.1 M HCHO in 10 mM KOH. 1 mM t-butanol was used as an internal standard and water suppression method was employed.

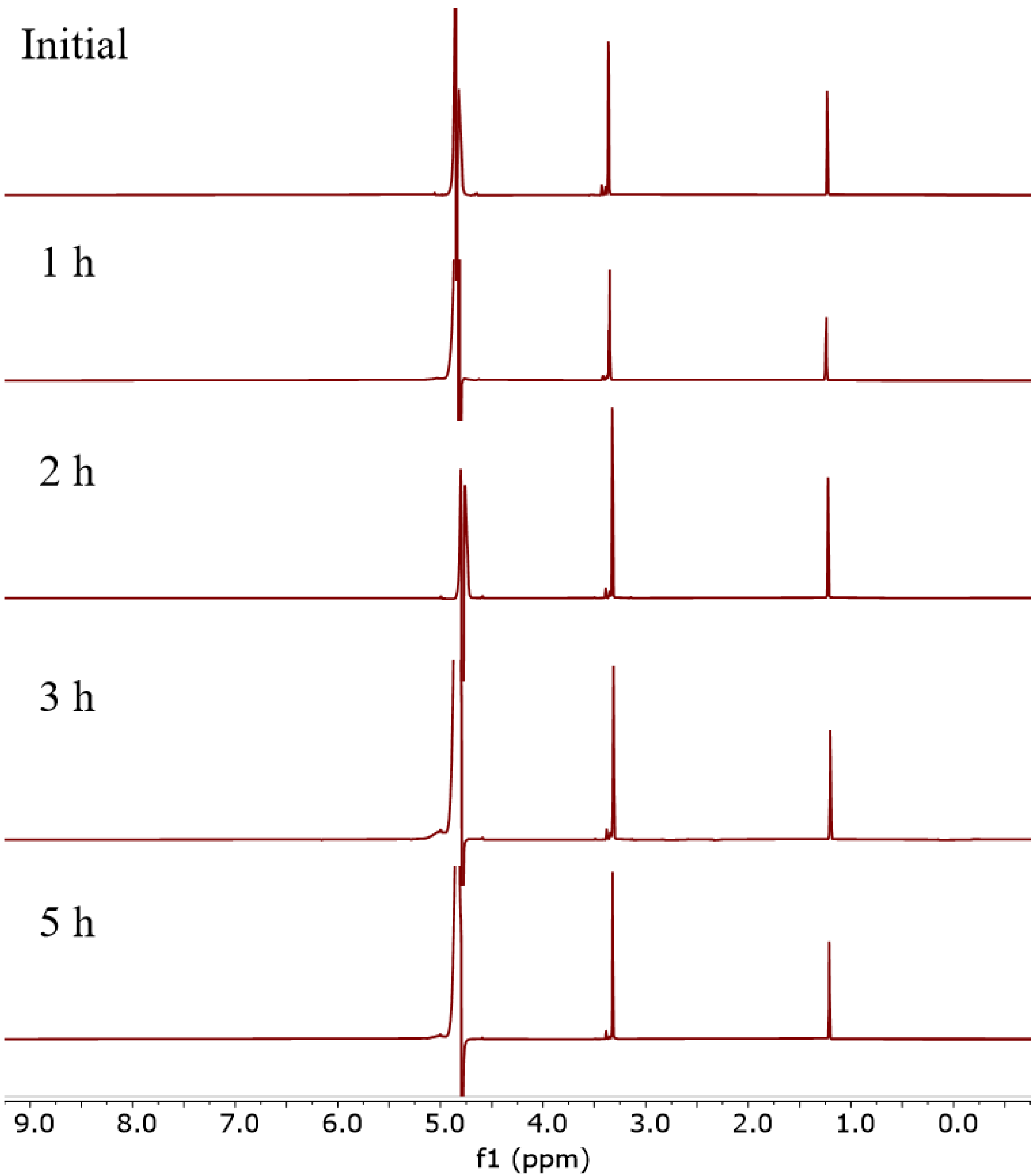

Figure S5. Time-dependent ^{1}H NMR spectra showing the Cannizzaro side reaction when dissolving 0.1 M HCHO in 0.1 M KOH. 1 mM t-butanol was used as an internal standard and water suppression method was employed.

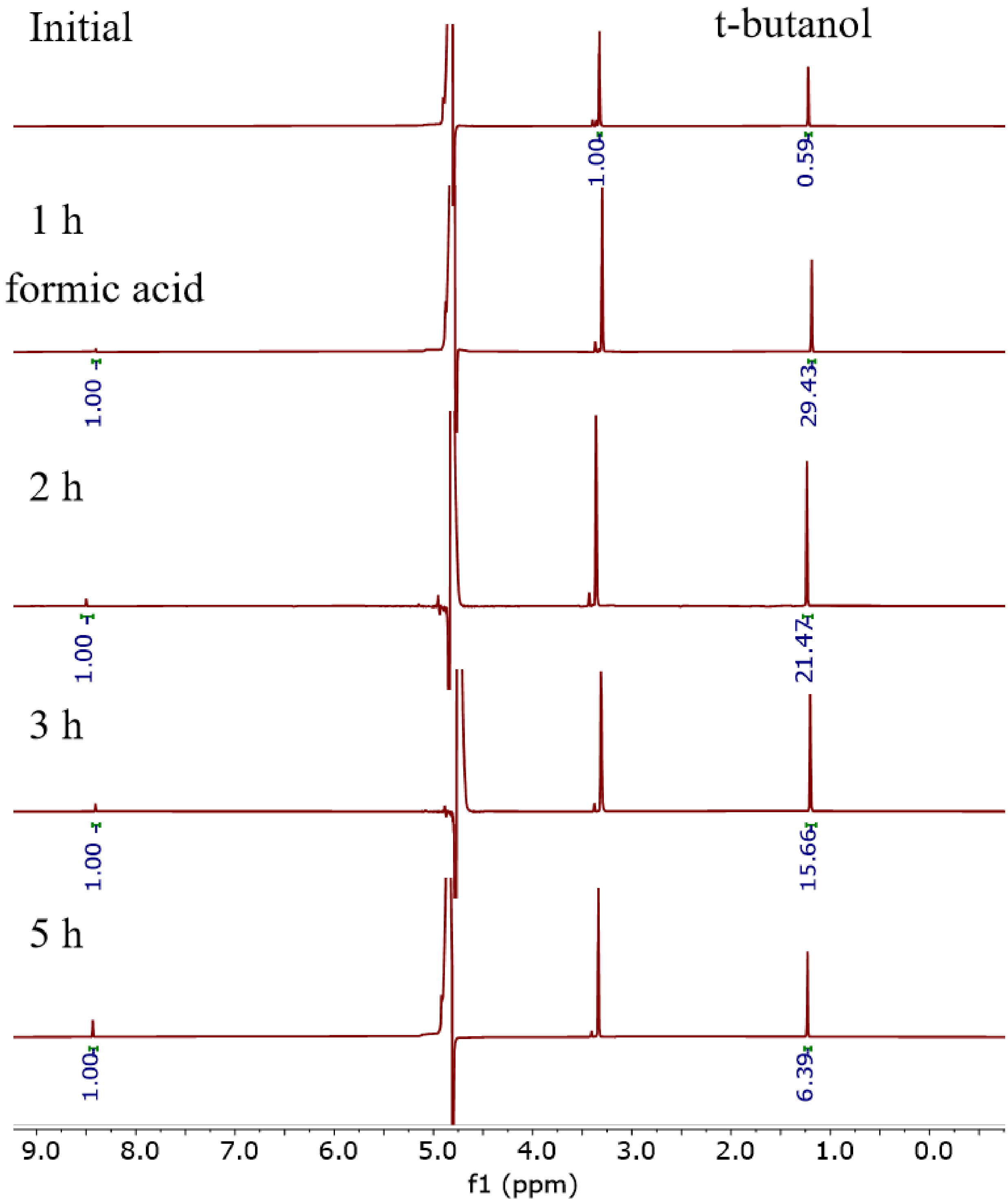

Figure S6. Time-dependent ^{1}H NMR spectra showing the Cannizzaro side reaction when dissolving 0.1 M HCHO in 1 M KOH. 1 mM t-butanol was used as an internal standard and water suppression method was employed.

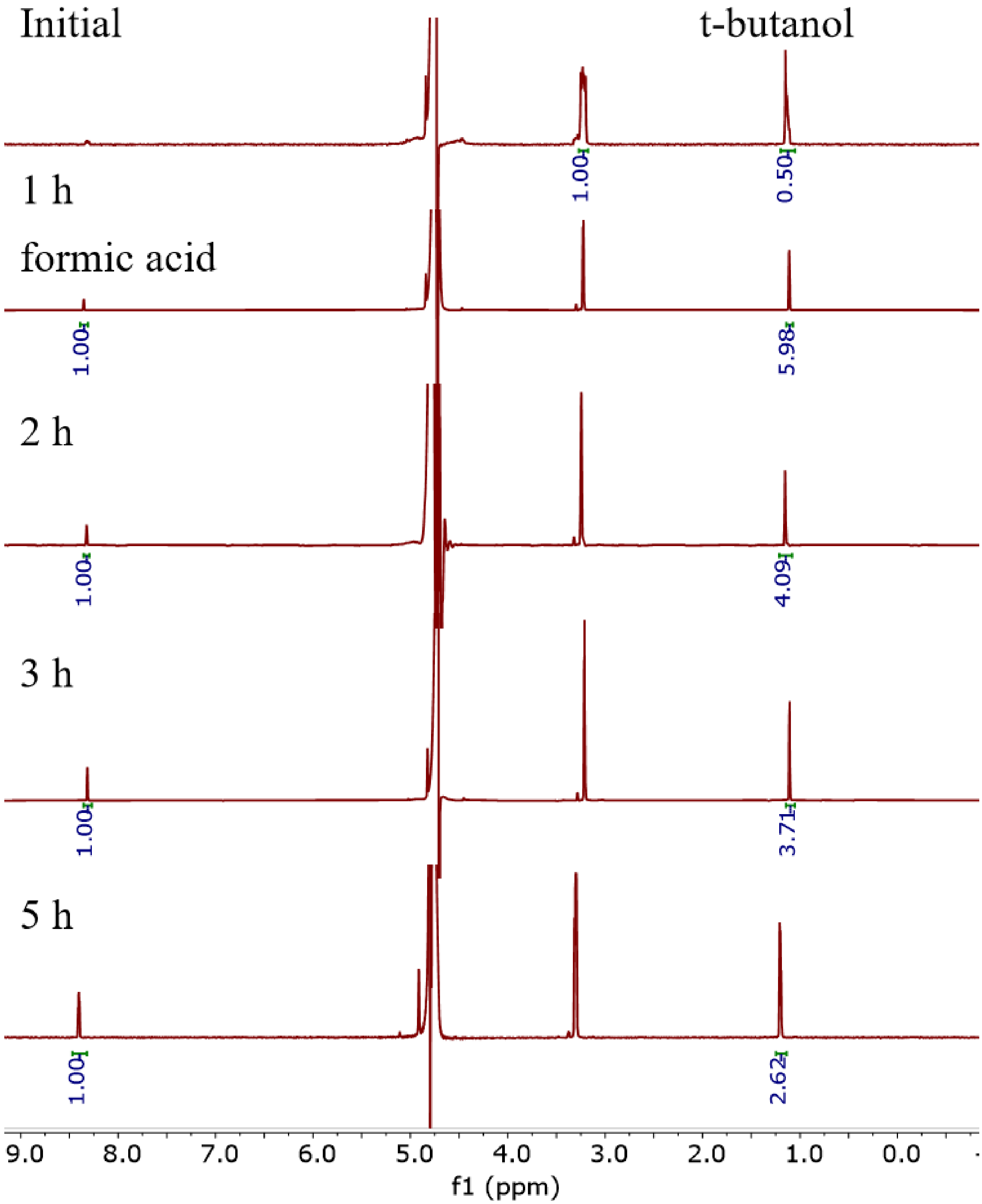

Figure S7. Time-dependent ^{1}H NMR spectra showing the Cannizzaro side reaction when dissolving 0.1 M HCHO in 2 M KOH. 1 mM t-butanol was used as an internal standard and water suppression method was employed.

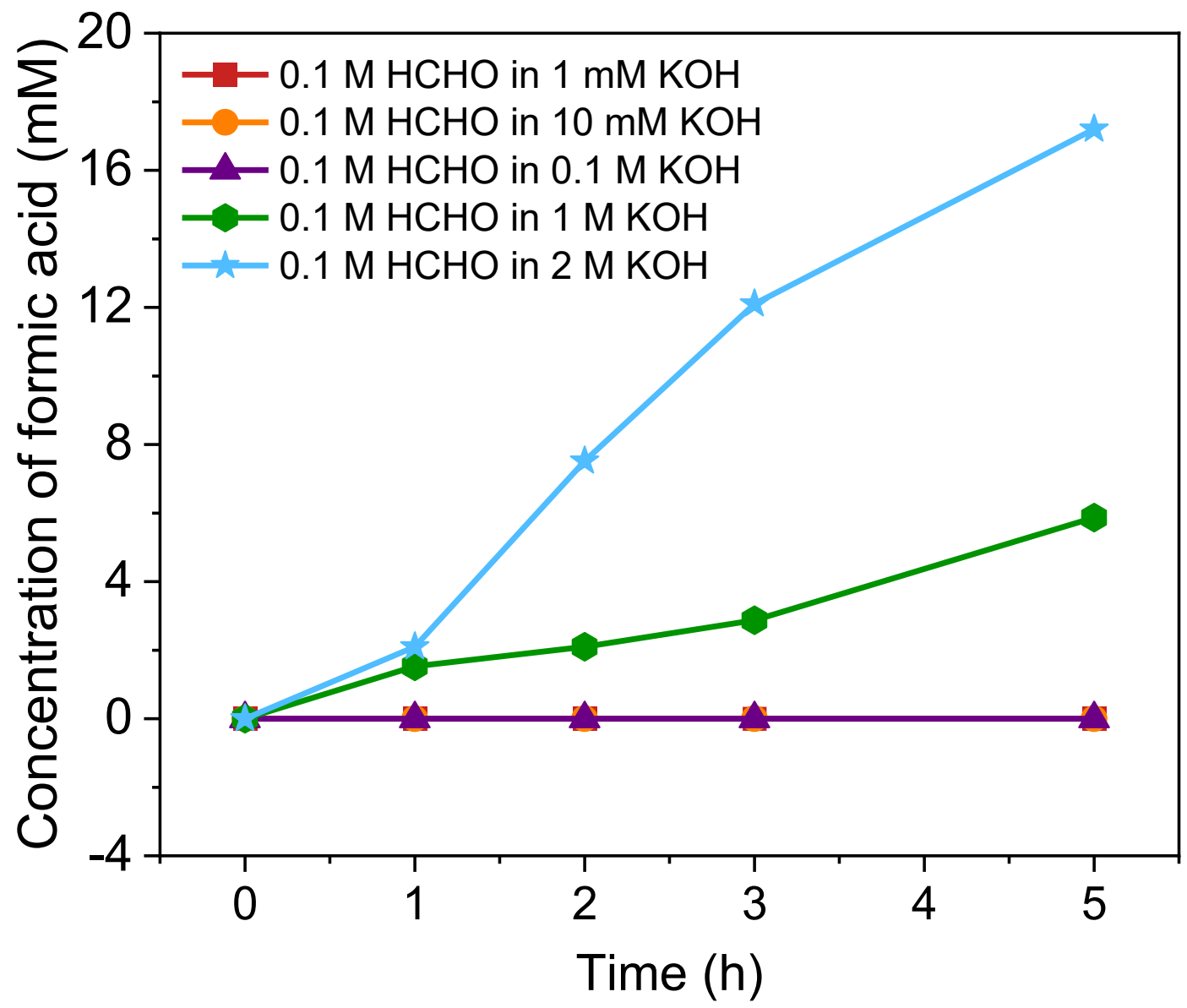

Figure S8. Direct plots generated based on collected ^{1}H NMR spectra showing the Cannizzaro side reaction when dissolving 0.1 M HCHO in different concentrations of KOH.

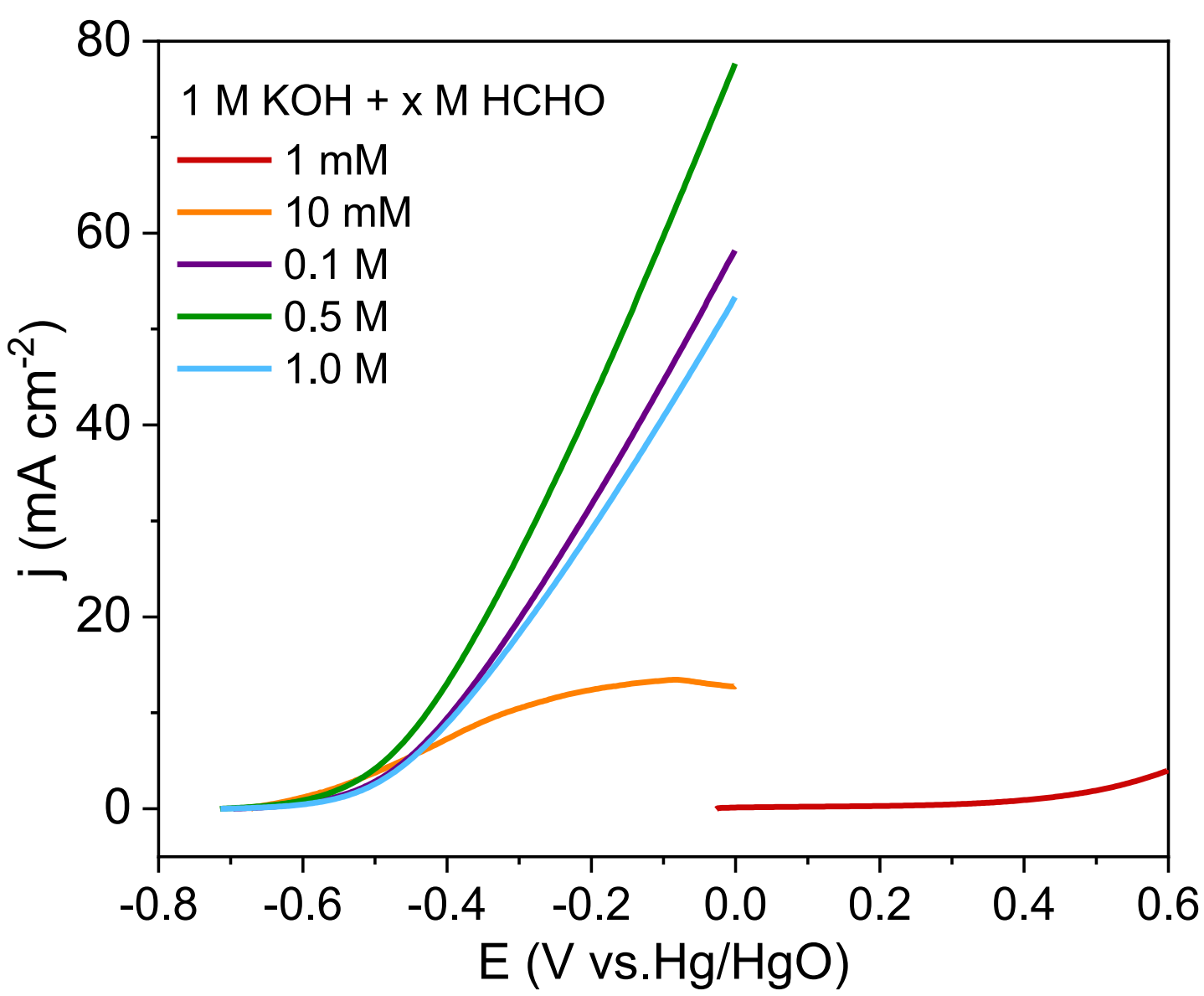

Figure S9. LSV curves recorded on Pd/RDE in Ar-saturated 1 M KOH with different concentrations of HCHO at 1500 rpm with a scan rate of 5 mV/s.

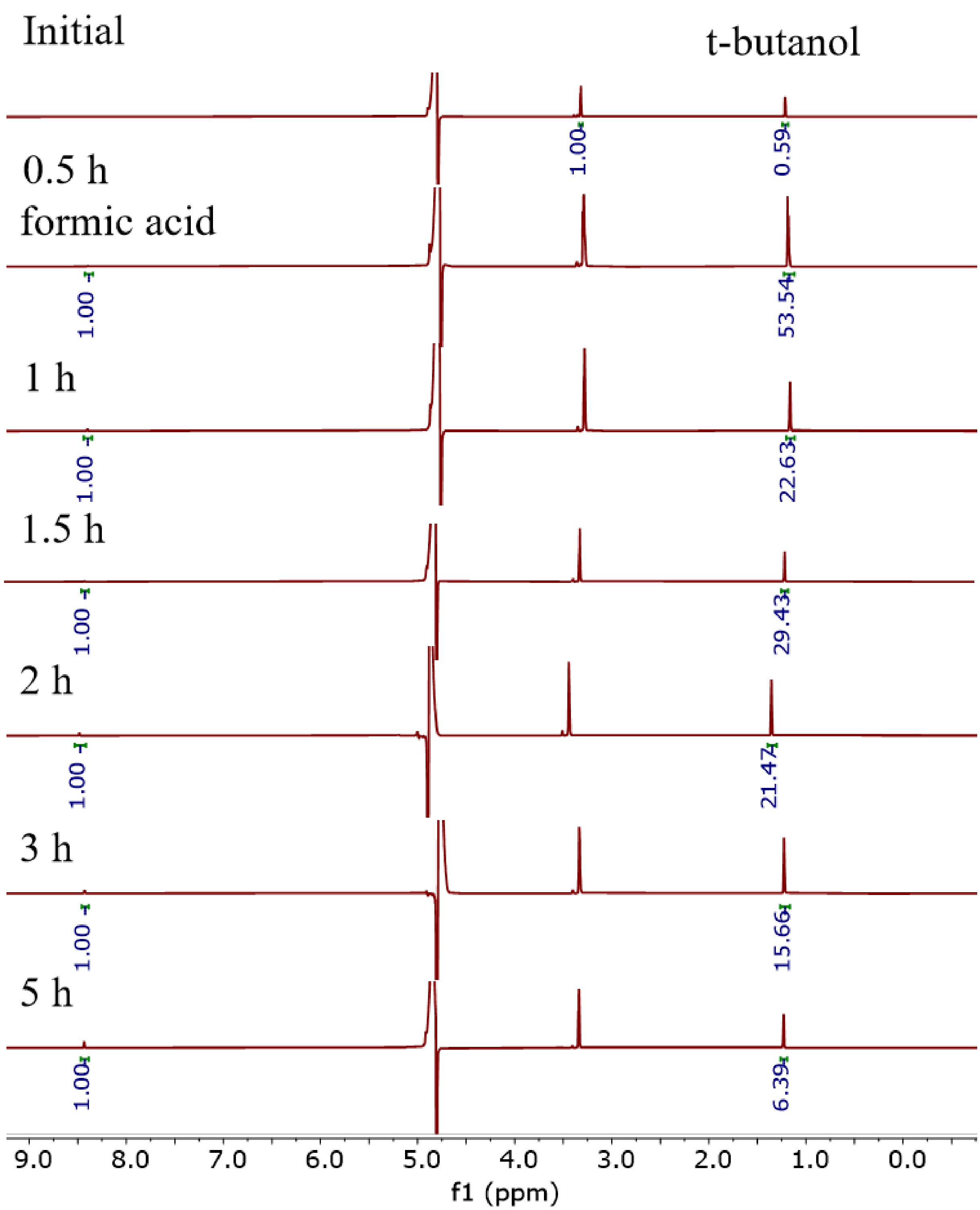

Figure S10. Time-dependent ^1H NMR spectra showing the Cannizzaro side rection when dissolving 0.1 M HCHO in 1 M KOH. 1 mM t-butanol was used as an internal standard and water suppression method was employed.

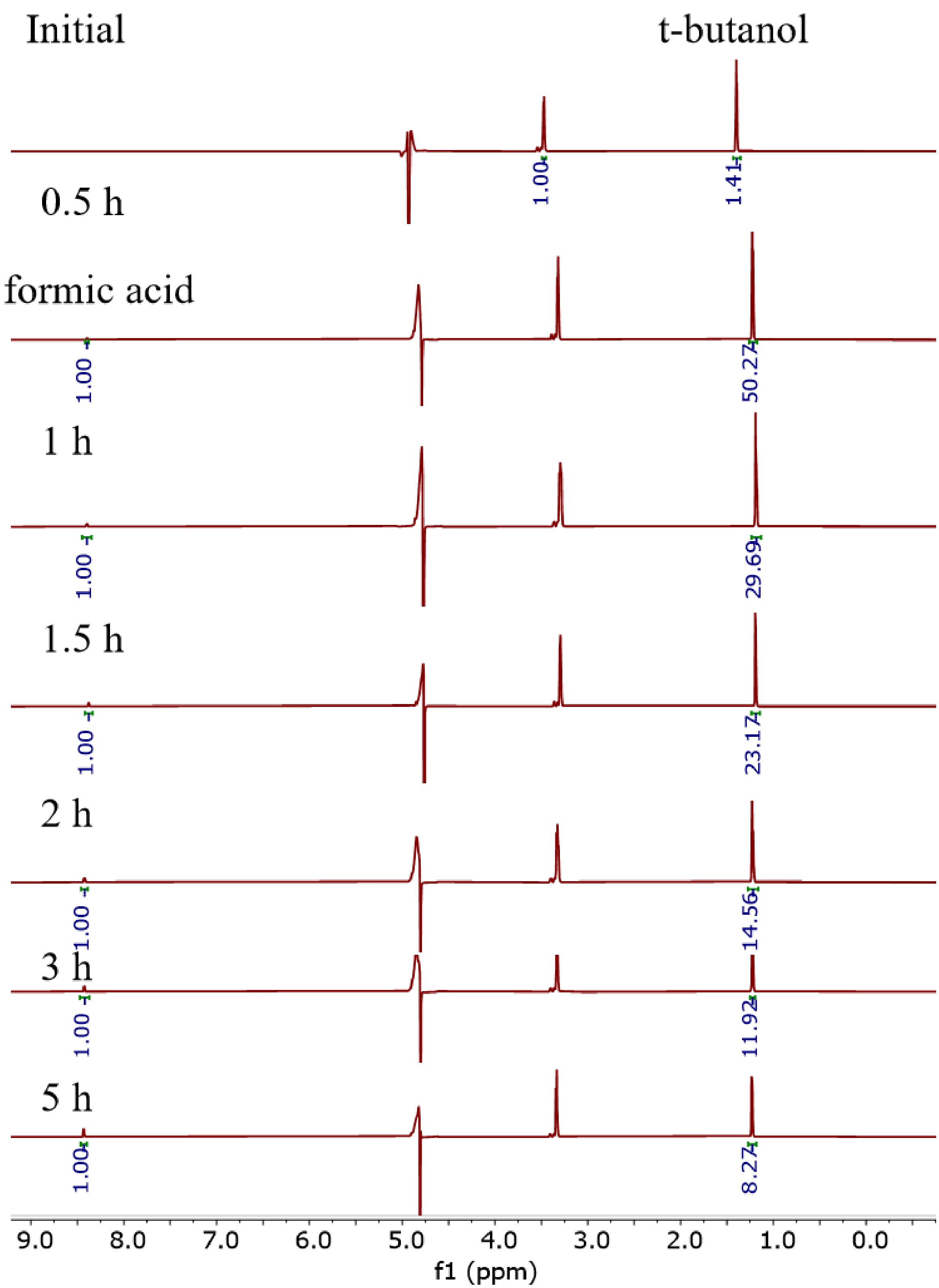

Figure S11. Time-dependent ^{1}H NMR spectra showing the Cannizzaro side reaction when dissolving 0.5 M HCHO in 1 M KOH. 10 mM t-butanol was used as an internal standard and water suppression method was employed.

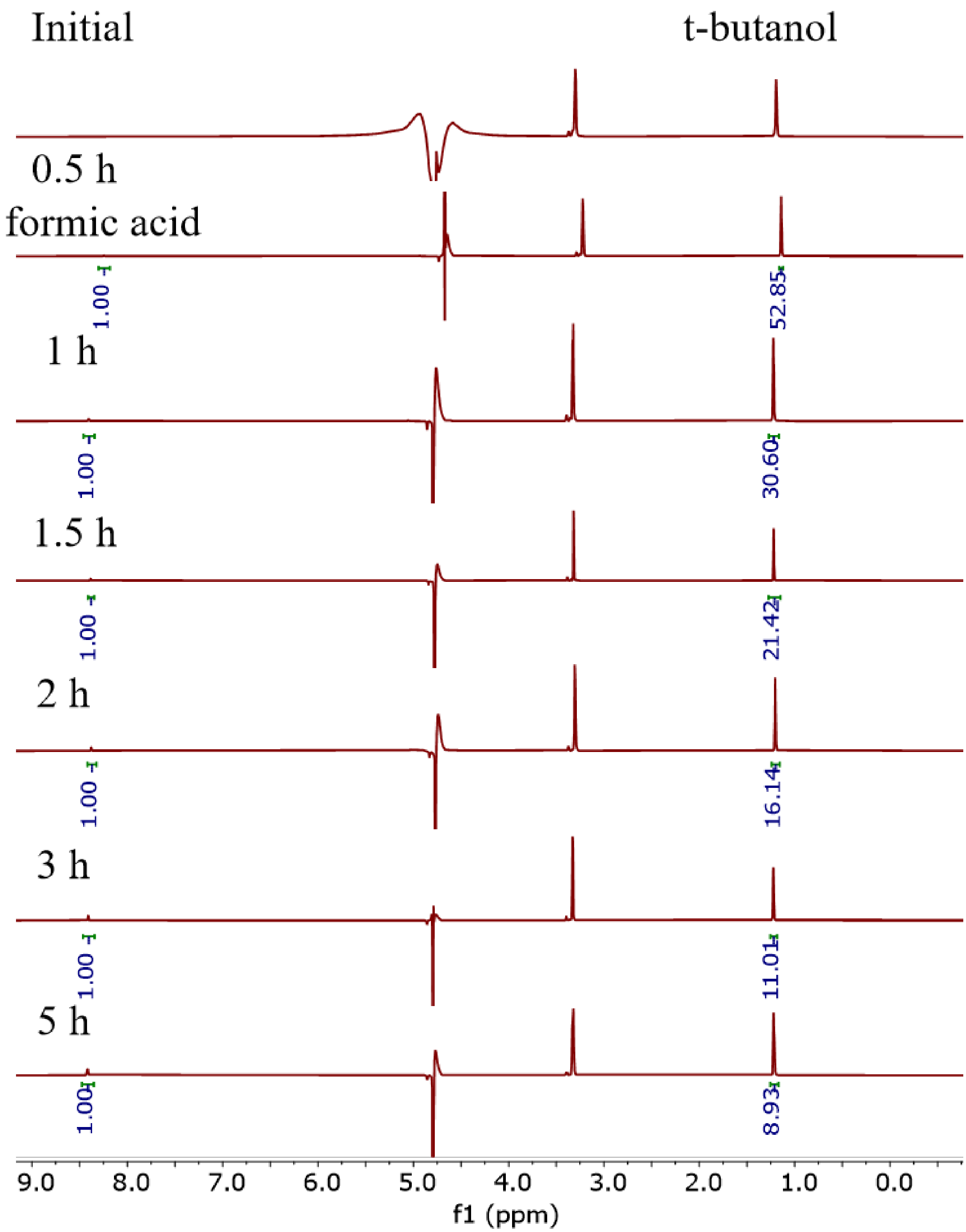

Figure S12. Time-dependent ^{1}H NMR spectra showing the Cannizzaro side reaction when dissolving 1 M HCHO in 1 M KOH. 10 mM t-butanol was used as an internal standard and water suppression method was employed.

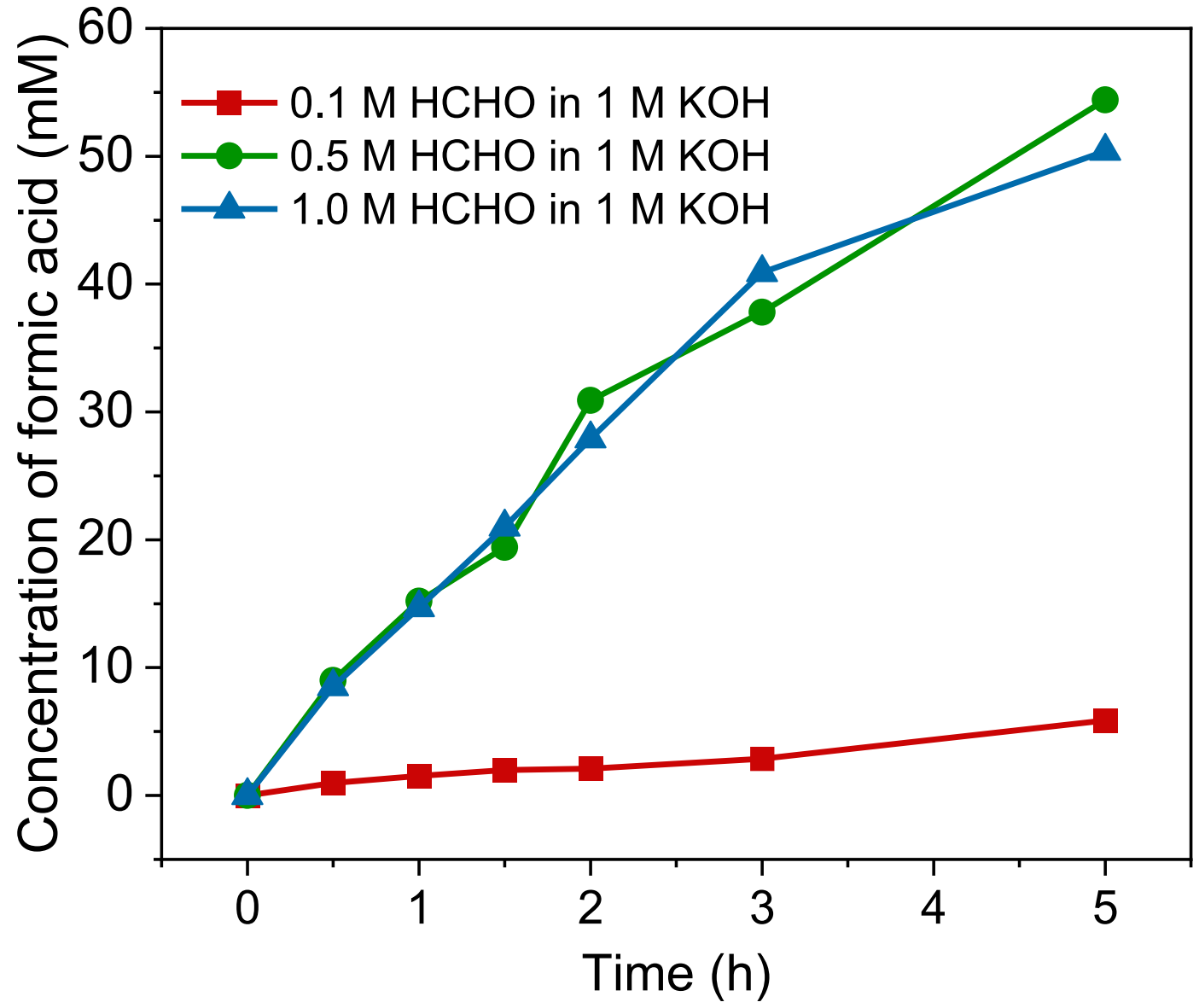

Figure S13. Direct plots generated based on collected ^{1}H NMR spectra showing the Cannizzaro side reaction when dissolving 0.1 M, 0.5 M and 1 M HCHO in 1 M KOH.

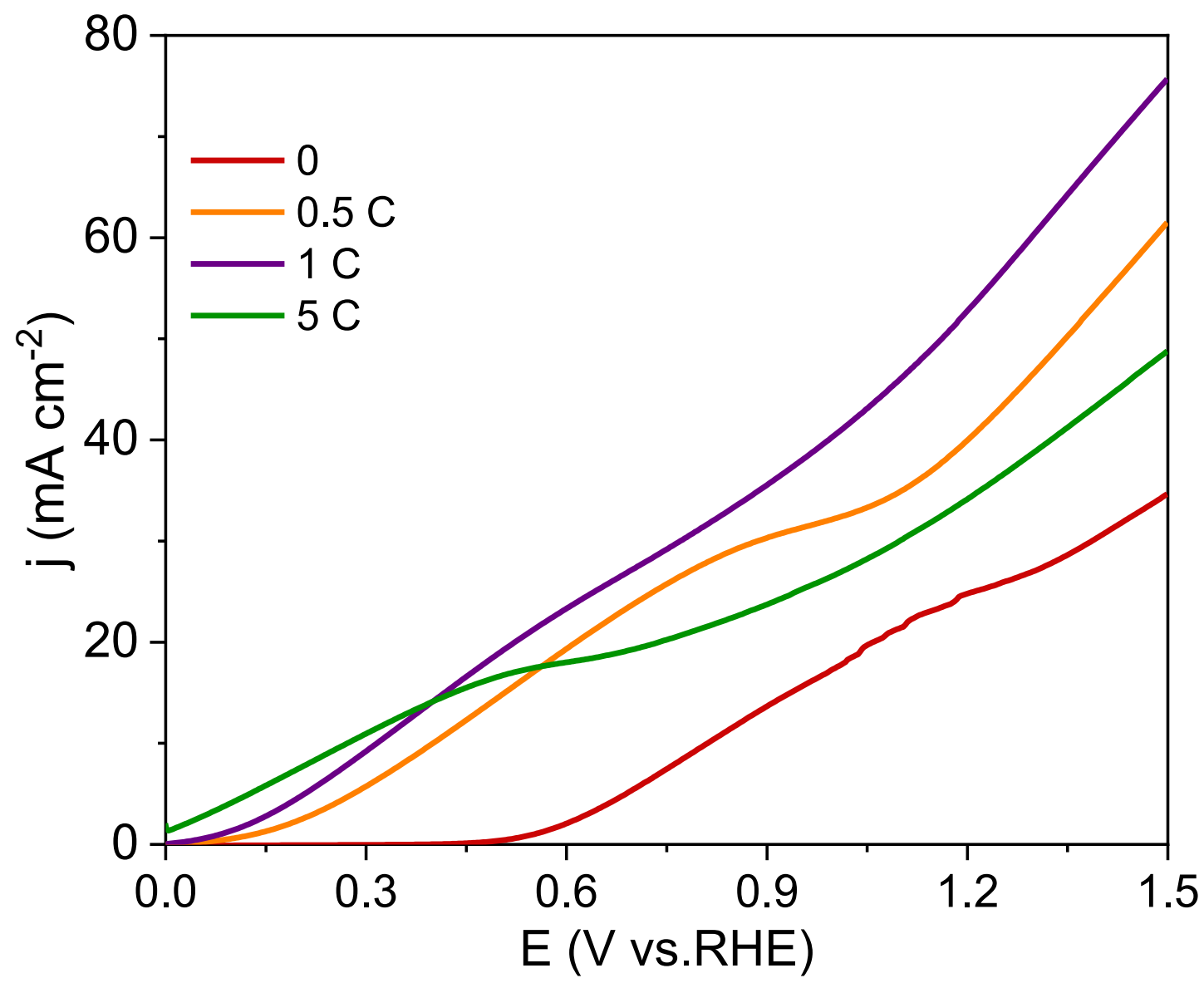

Figure S14. LSV curves recorded on J-Pdm in 1 M KOH with 0.5 M HCHO at a scan rate of 10 mV/s when the membrane surface facing the FOR chamber was modified by electrodeposition of different charges of Pd nanoparticles.

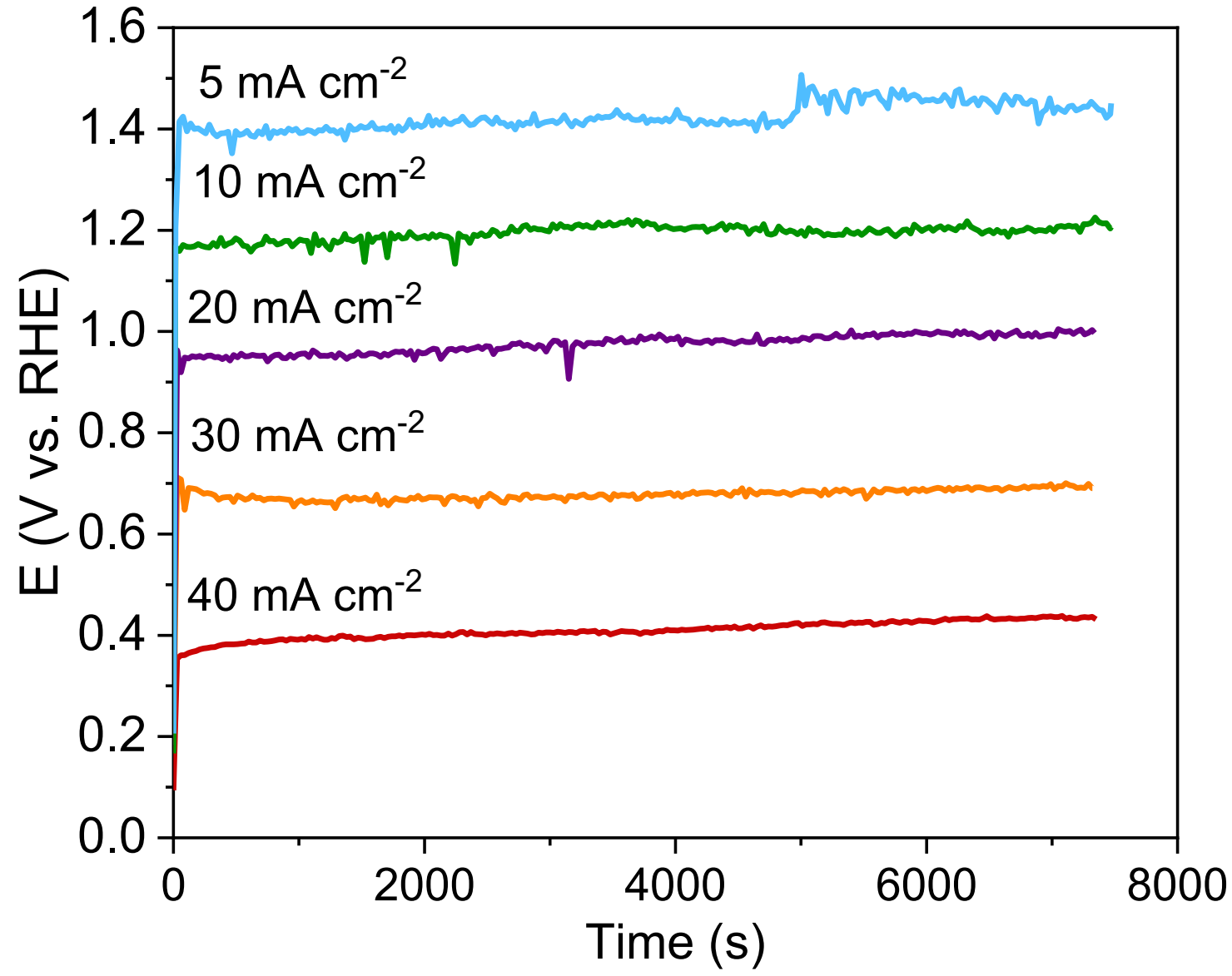

Figure S15. E-t curves when different current densities were applied in the FOR chamber.

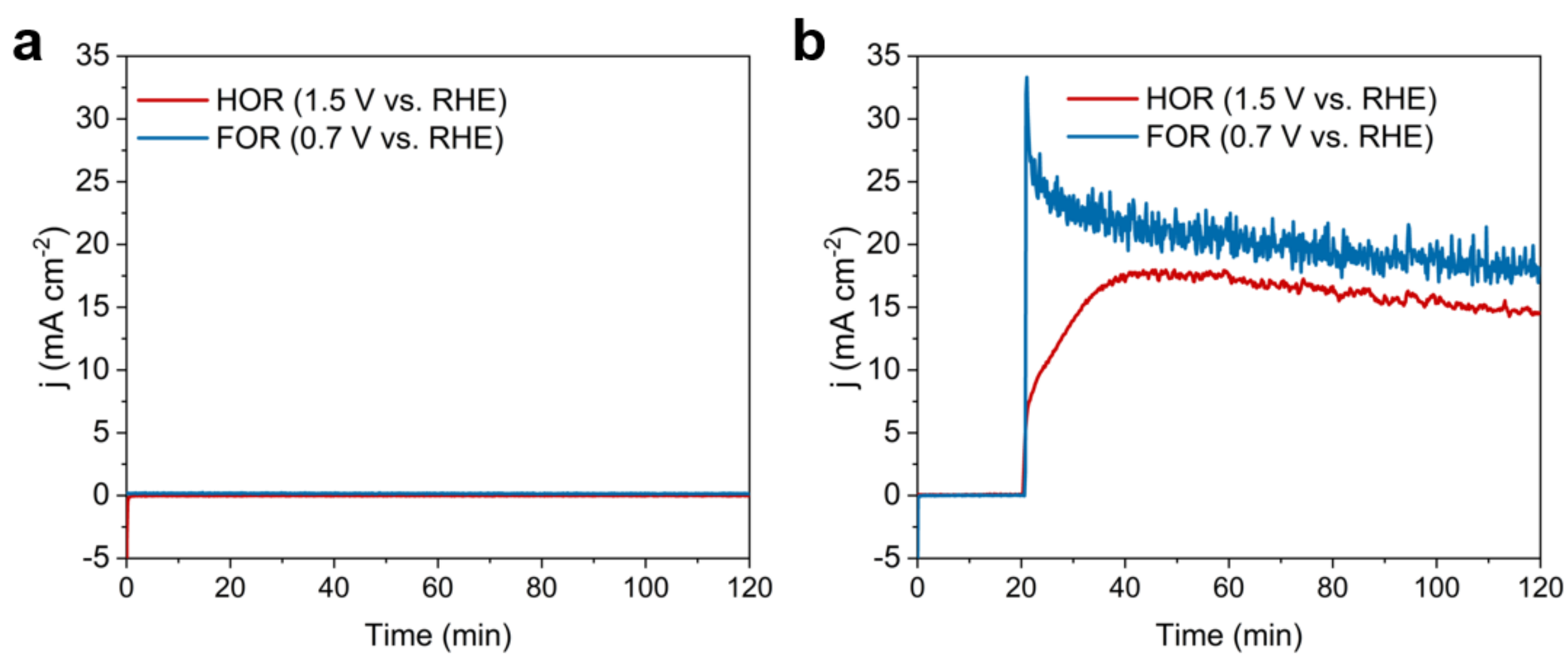

Figure S16. Control experiments verifying the FOR-dependent origin of the HOR current. (**a**) Chronoamperometric responses in the FOR and HOR chambers in the absence of HCHO, with the FOR chamber and the HOR chamber maintained at 0.7 and 1.5 V vs RHE, respectively. (**b**) Chronoamperometric responses under the same potential conditions before and after the addition of HCHO. Both currents remained negligible during the initial 20 min in the absence of HCHO. Upon injection of 0.5 M HCHO into the FOR chamber, the FOR current appeared immediately and was accompanied by the development of a substantial HOR current at the opposite side of the Pd membrane.

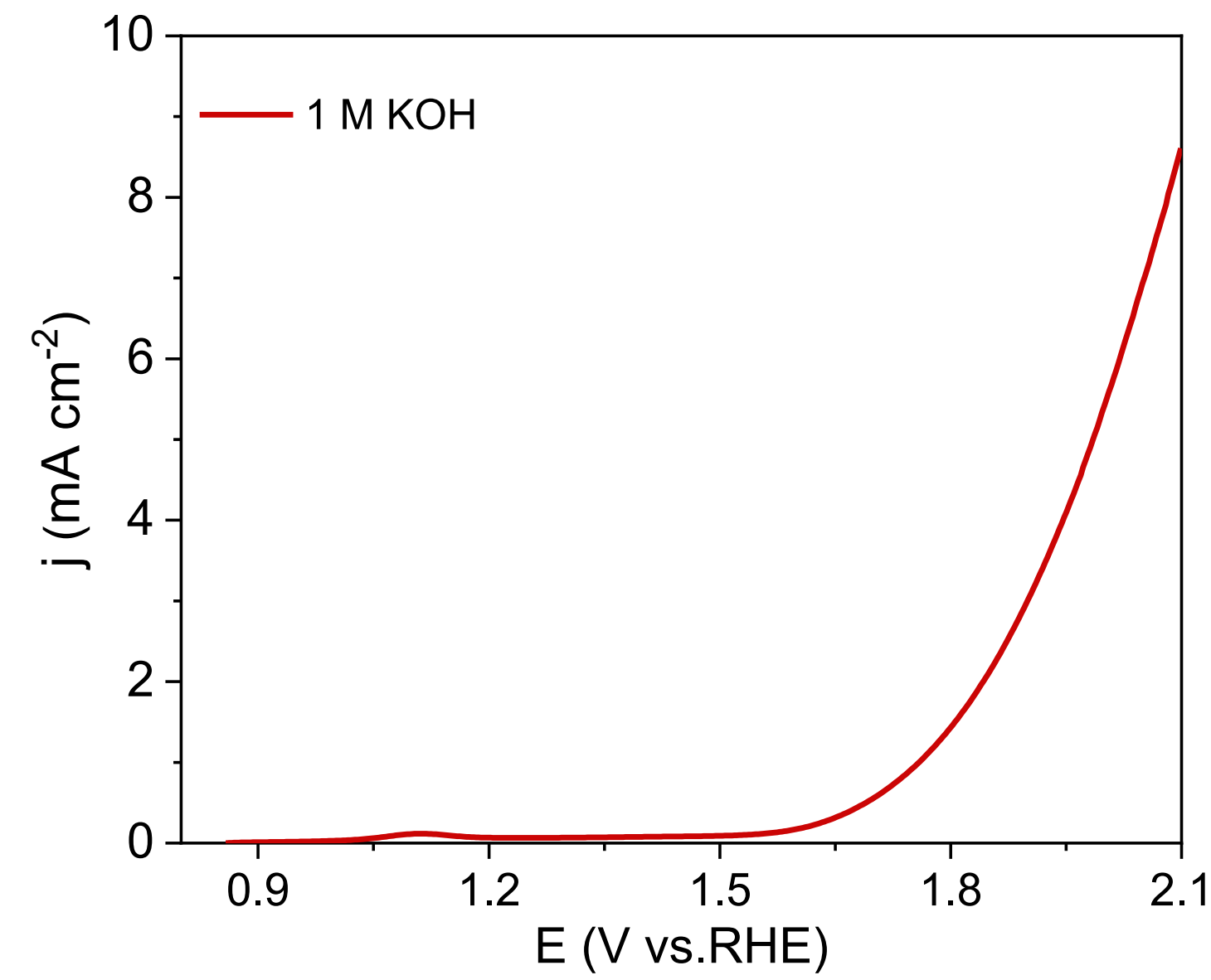

Figure S17. LSV curve for OER on Pd_m in 1 M KOH at a scan rate of 10 mV/s.

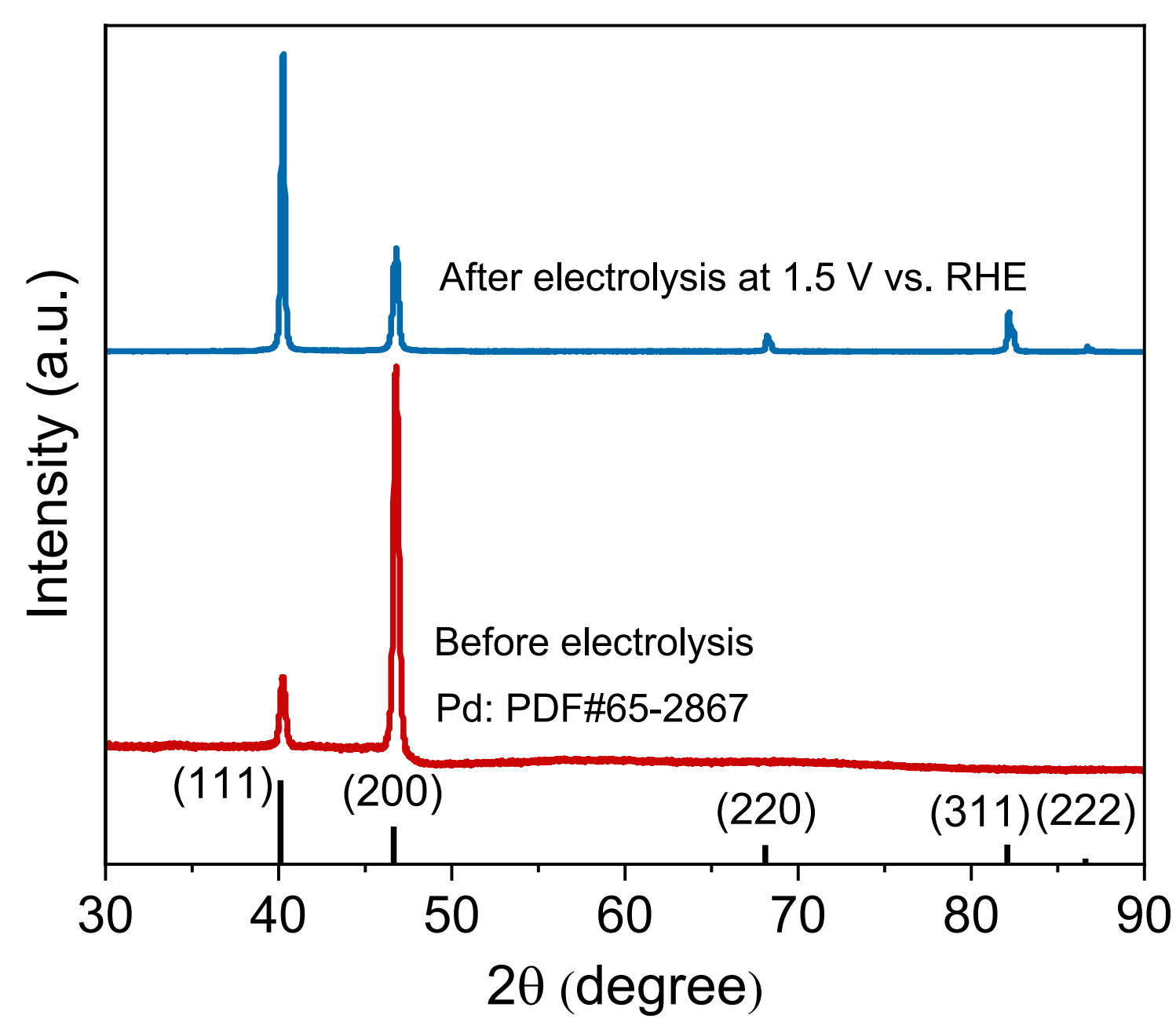

Figure S18. XRD patterns of HOR-facing Pd_m before and after electrolysis at 1.5 V vs RHE.

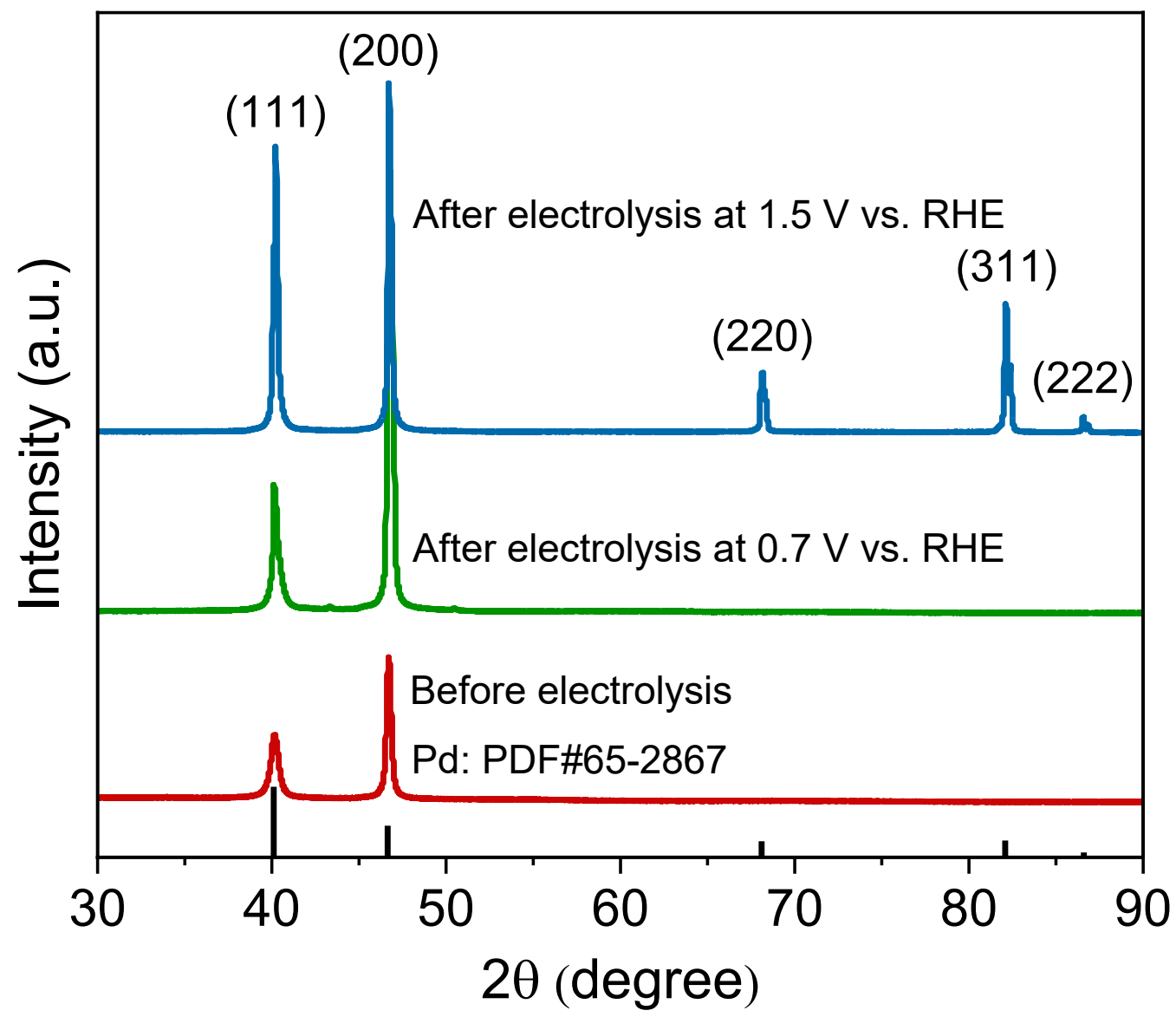

Figure S19. XRD patterns of FOR-facing Pd_m (electrodeposited with 1 C of Pd nanoparticles) before and after electrolysis at 0.7 and 1.5 V vs RHE.

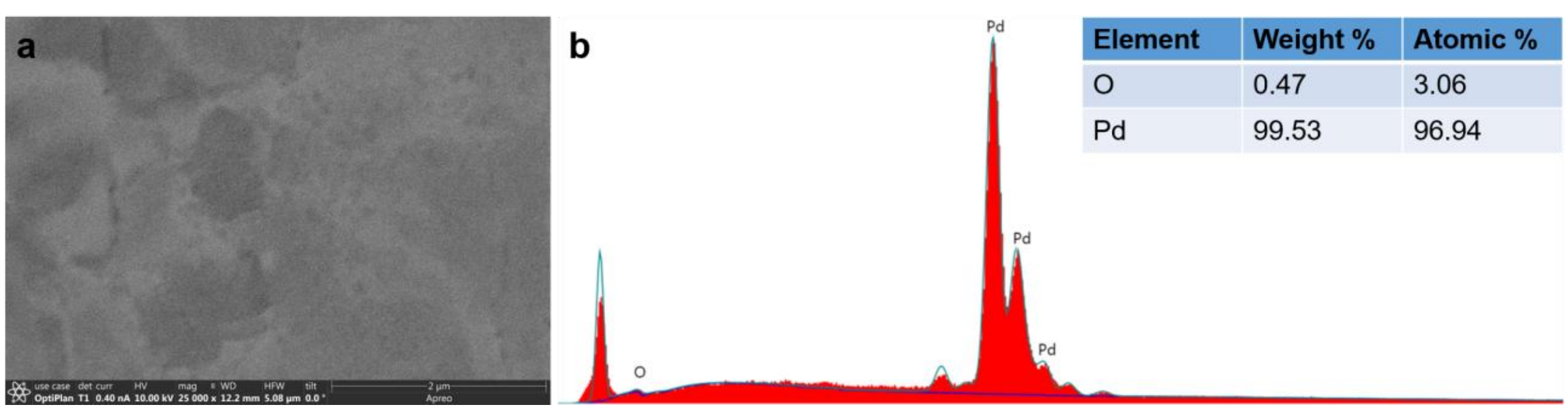

Figure S20. SEM image (**a**) and its corresponding EDS characterization (**b**) of HOR-facing Pd_m before electrolysis.

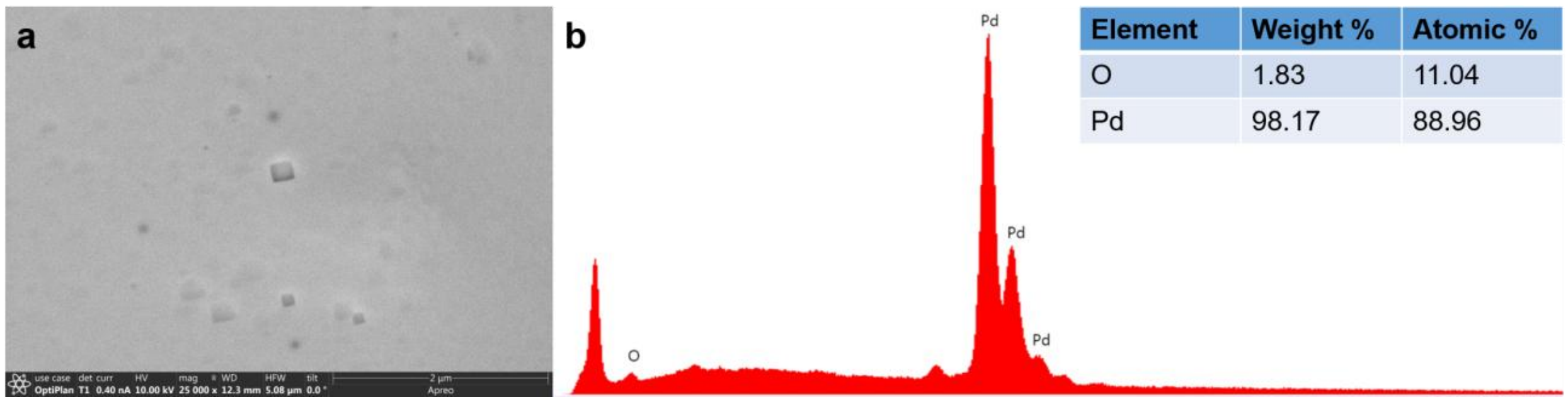

Element	Weight %	Atomic %
O	1.83	11.04
Pd	98.17	88.96

Figure S21. SEM image (**a**) and its corresponding EDS characterization (**b**) of HOR-facing Pd_m after electrolysis at 1.5 V vs RHE.

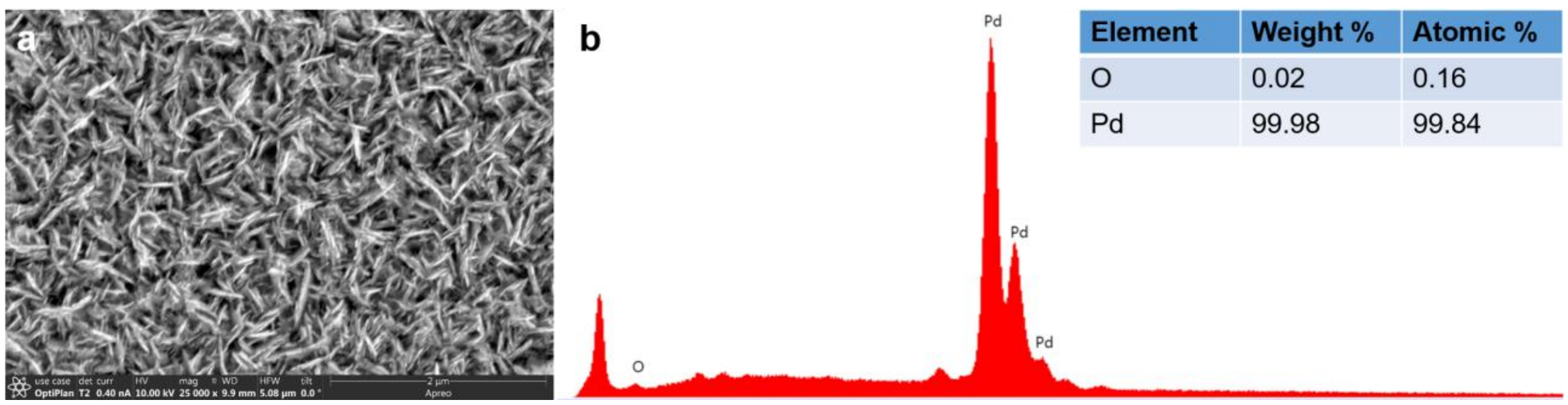

Element	Weight %	Atomic %
O	0.02	0.16
Pd	99.98	99.84

Figure S22. SEM image (**a**) and its corresponding EDS characterization (**b**) of FOR-facing Pd_m (electrodeposited with 1 C of Pd nanoparticles) before electrolysis.

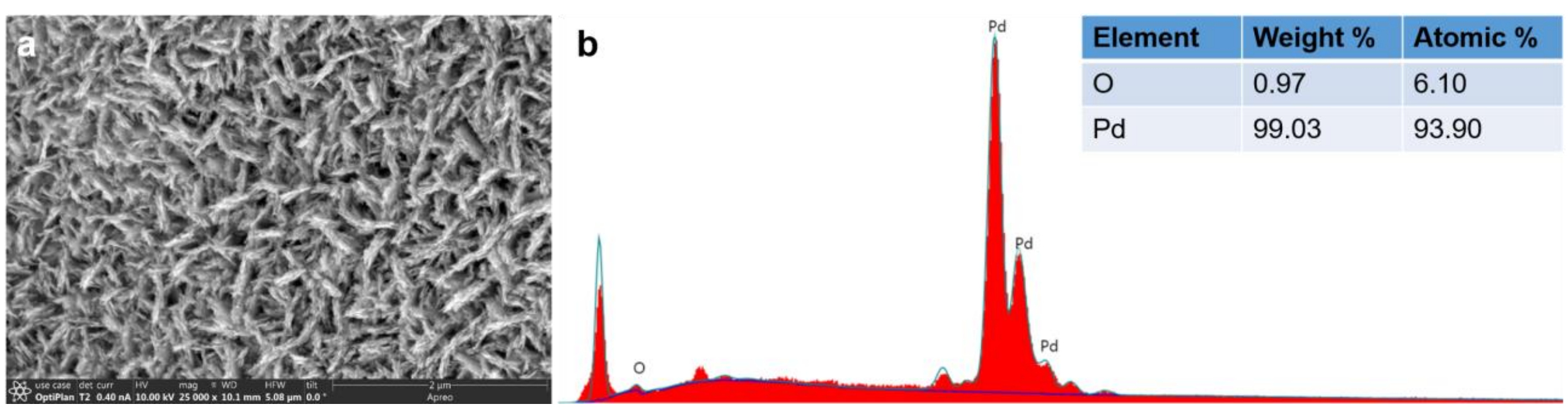

Element	Weight %	Atomic %
O	0.97	6.10
Pd	99.03	93.90

Figure S23. SEM image (**a**) and its corresponding EDS characterization (**b**) of FOR-facing Pd_m (electrodeposited with 1 C of Pd nanoparticles) after electrolysis at 0.7 V vs RHE.

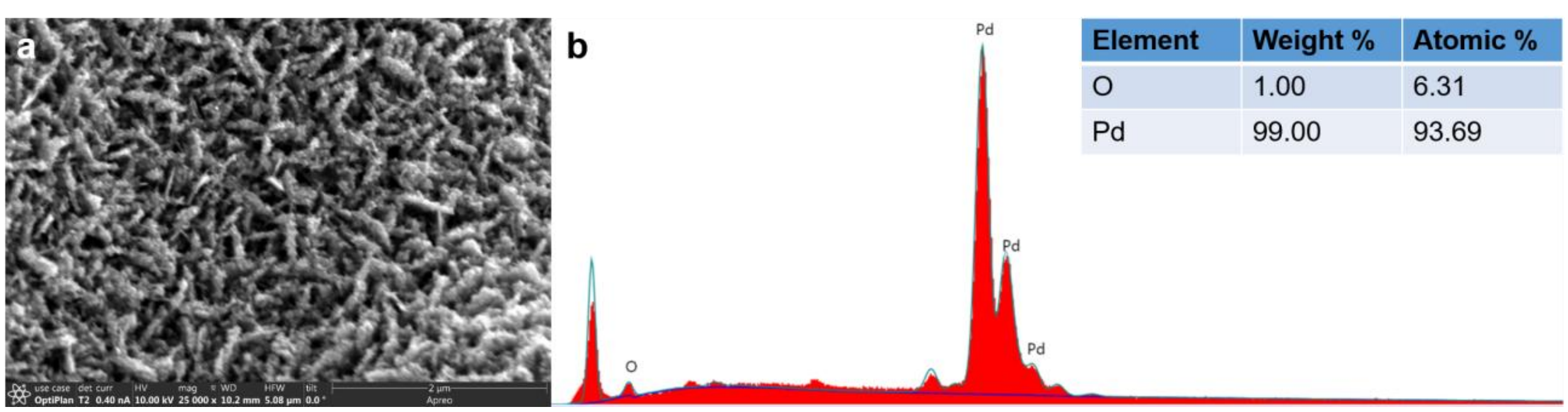

Element	Weight %	Atomic %
O	1.00	6.31
Pd	99.00	93.69

Figure S24. SEM image (**a**) and its corresponding EDS characterization (**b**) of FOR-facing Pd_m (electrodeposited with 1 C of Pd nanoparticles) after electrolysis at 1.5 V vs RHE.

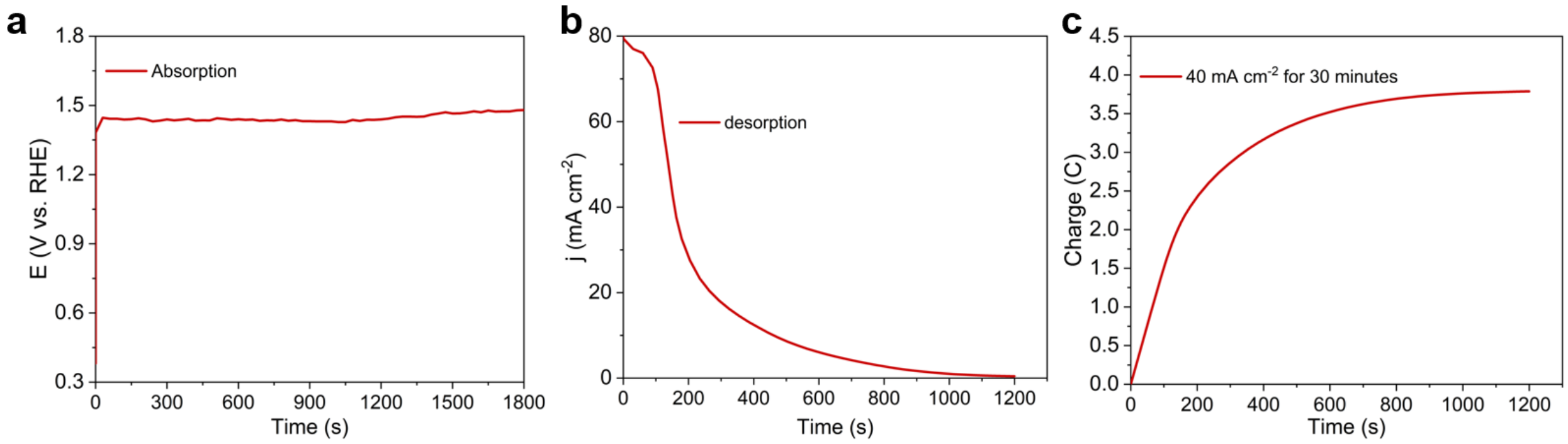

Figure S25. Three-step measurements for quantification of H/Pd ratio. (a) Representative E-t curve shows the constant current electrolysis for hydrogen loading. (b) I-t curves at 1.5 V vs. RHE shows the hydrogen desorption from Pd lattice and (c) the corresponding charge during desorption process. 0.5 M H_2SO_4 was used in the HOR chamber. For the H: Pd measurements, a 3-step workflow was employed to collect each data point. First, a constant current electrolysis was applied to the electrode to drive hydrogen absorption into the palladium lattice. Next, 1.5 V vs. RHE was applied, which oxidized hydrogen absorbed in the lattice back into protons, while the amount of charge passed during this step was measured. Lastly, the total current passed during this oxidation step was integrated to yield the total charge associated with the oxidation of adsorbed hydrogen. All H: Pd absorption values were determined by converting the oxidative (desorption) charge to moles using Faraday's constant. The moles of palladium in the sample divided the absorbed moles of hydrogen. The moles of palladium were calculated from the dry mass of the palladium sample.

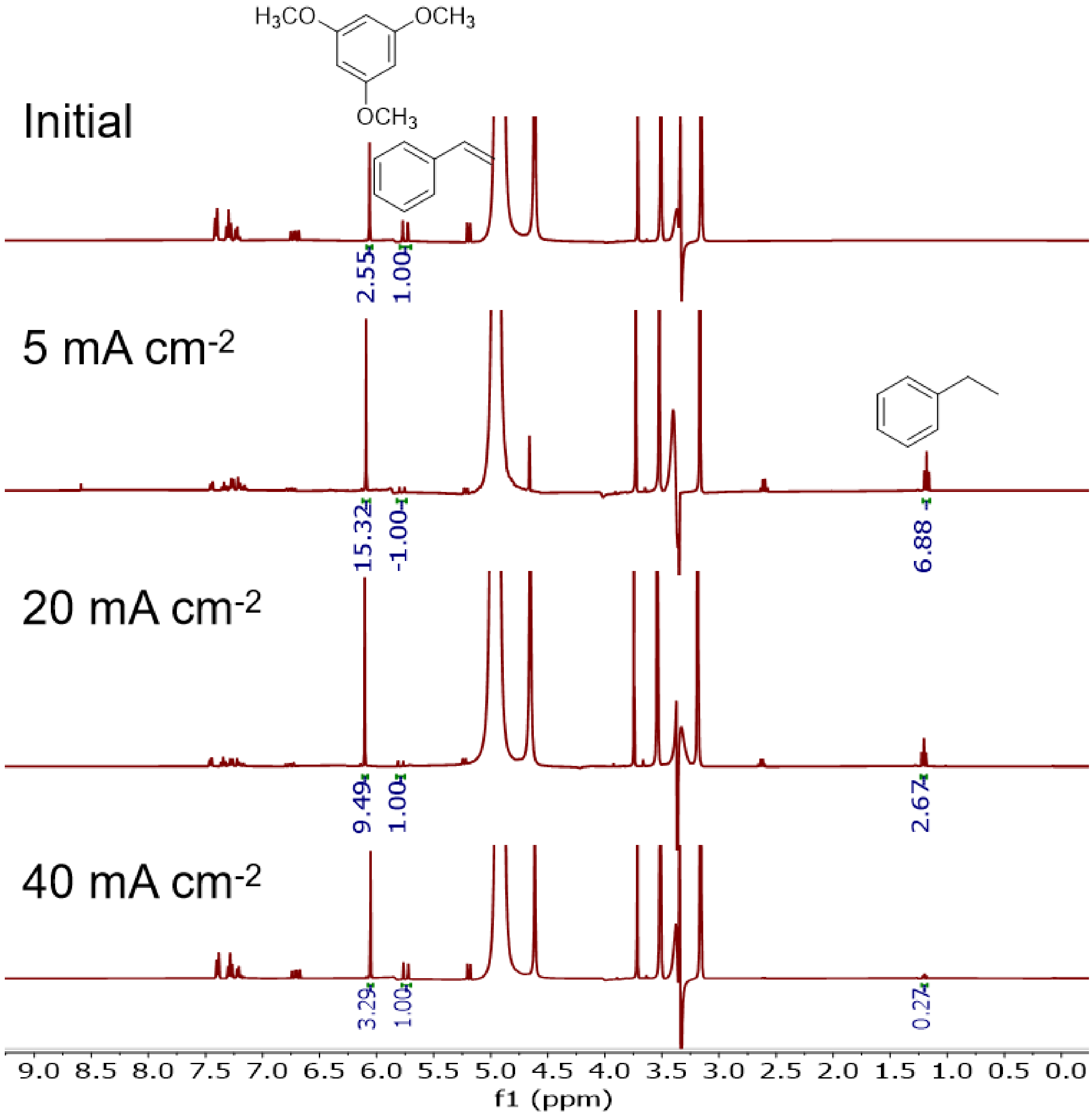

Figure S26. Representative ^{1}H NMR spectra of the hydrogenation of 50 mM styrene in 3 mL MeOH. The spectra were collected after each constant-current electrolysis followed by 2-h additional stirring. The concentration of ethylbenzene was determined by ^{1}H NMR using water suppression method using 10 mM 1,3,5-trimethoxybenzene as an internal standard.

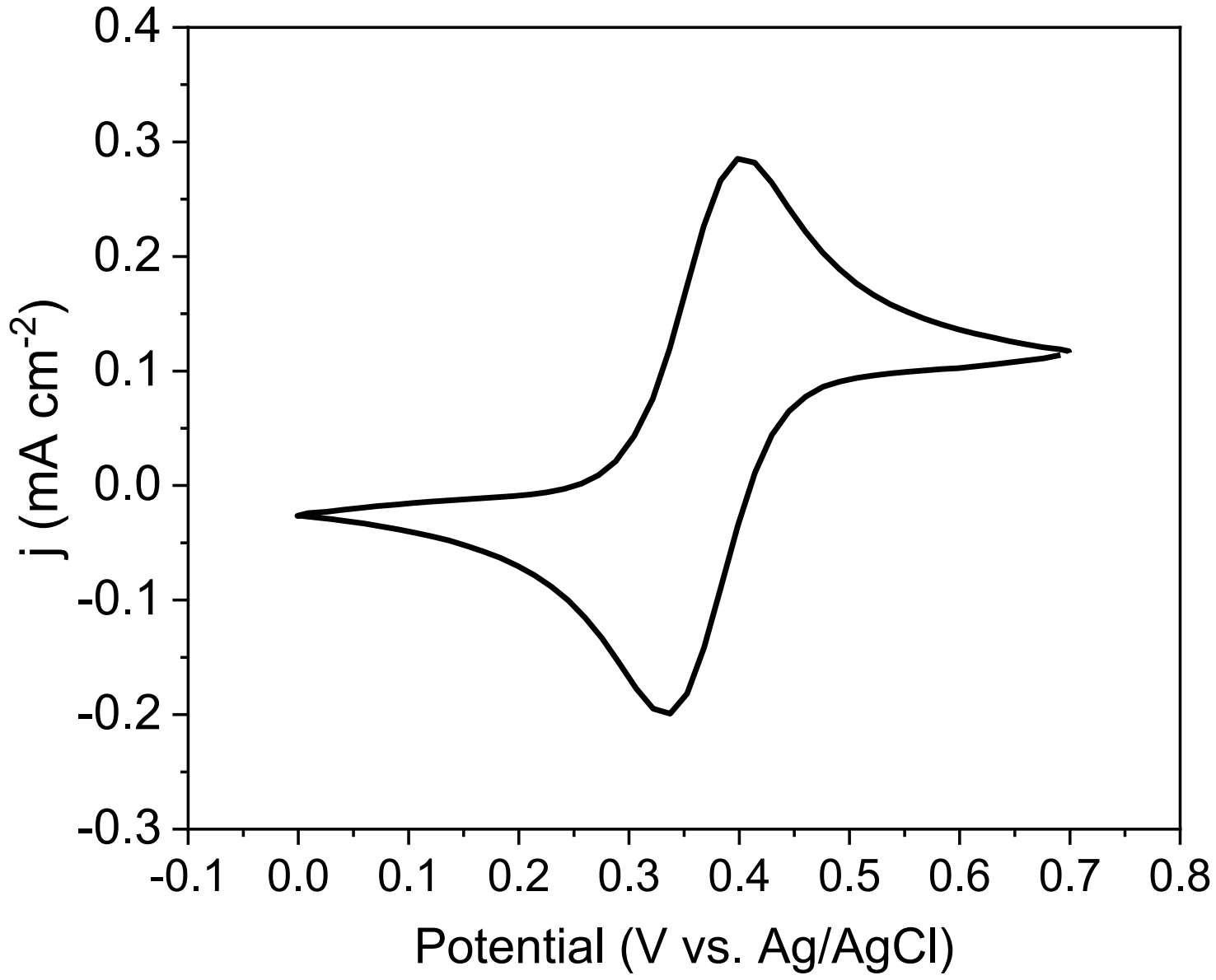

Figure S27. Cyclic voltammogram of ferrocene (Fc) collected at a scan rate of 100 mV/s with the reference electrode of Ag/AgCl.

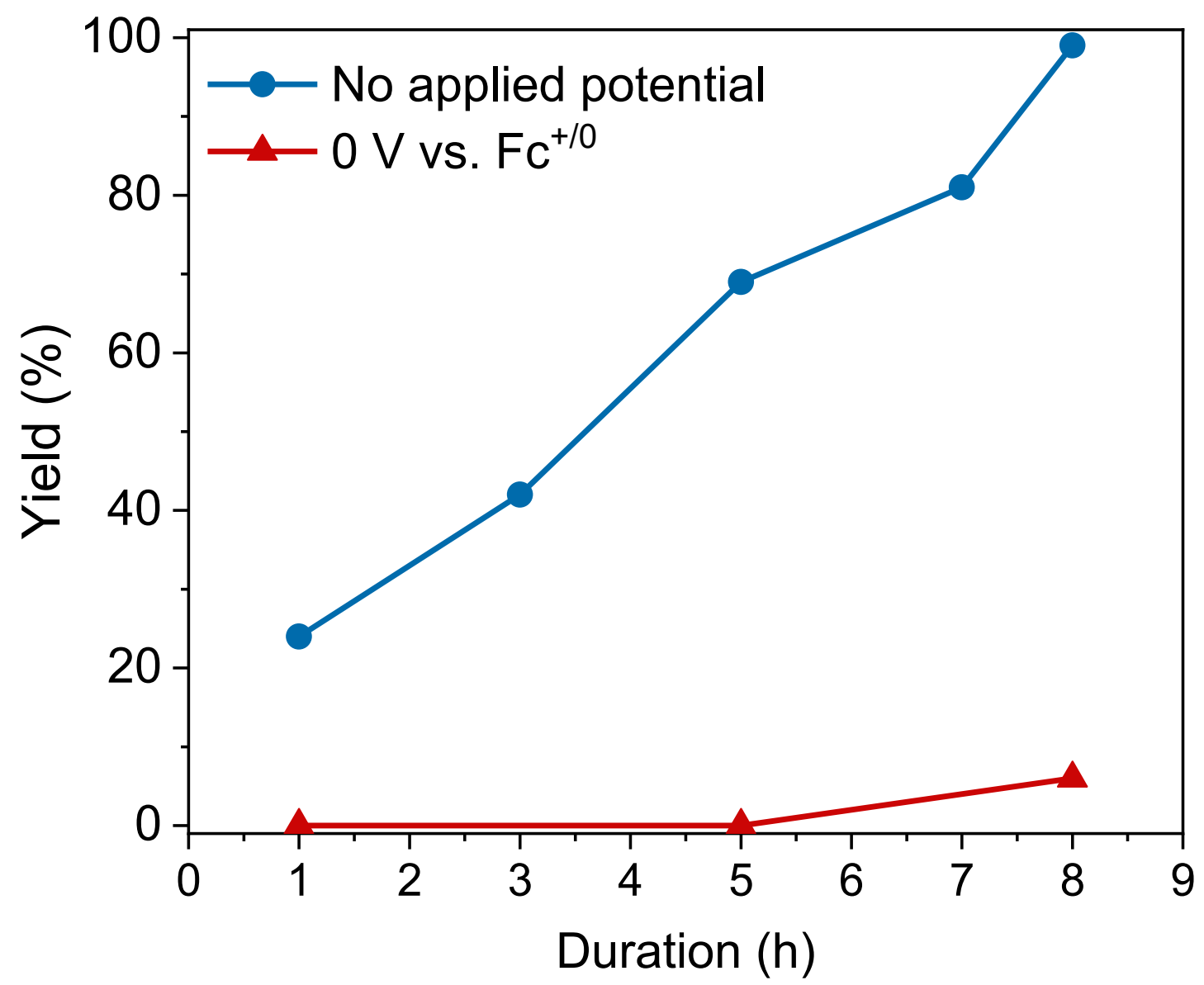

Figure S28. Time-resolved conversion profiles for styrene hydrogenation during 8-h electrolysis under two different conditions: (1) 0 V vs $Fc^{+/0}$ was applied in the chemical chamber; (2) no applied potential in the chemical chamber.

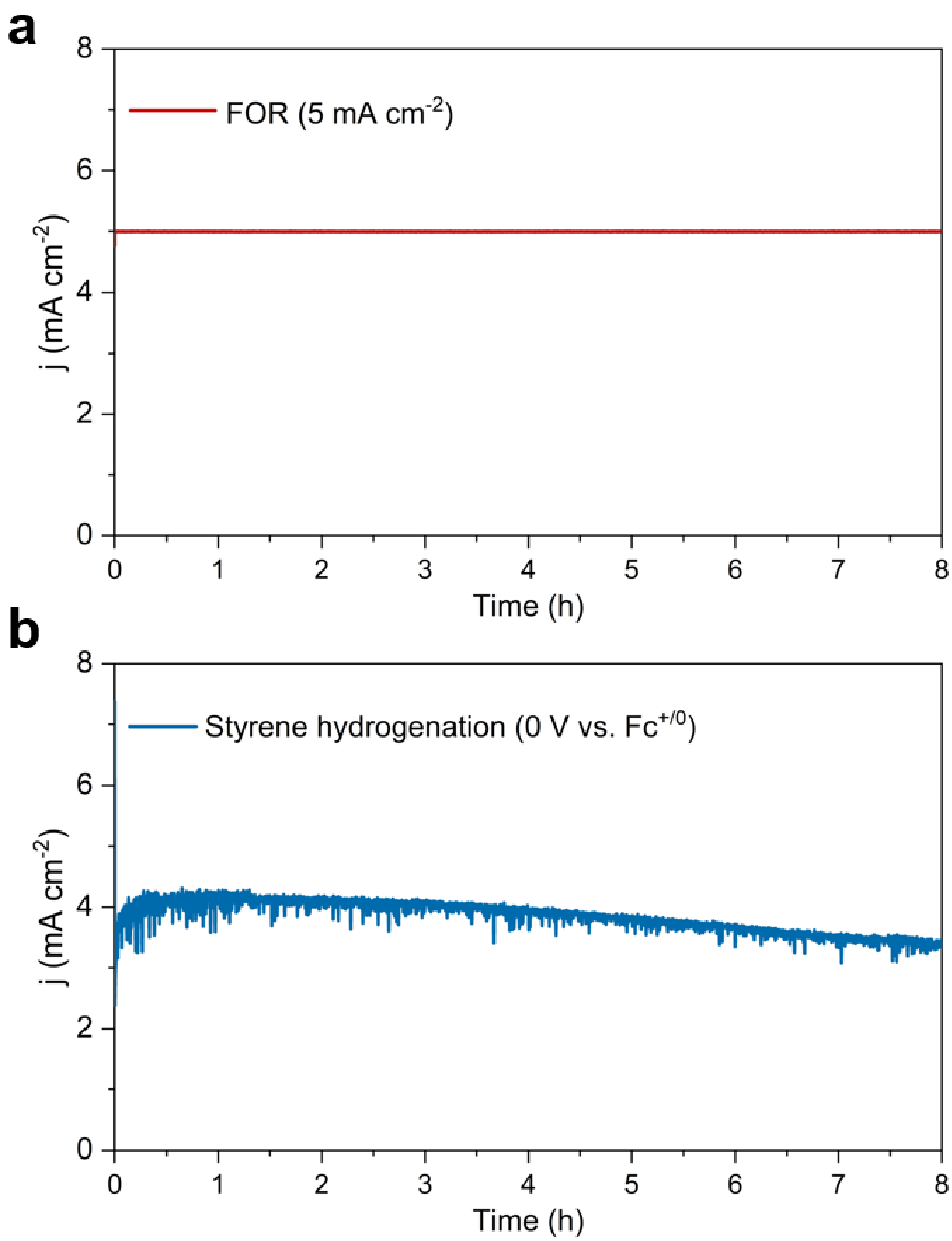

Figure S29. (**a**) Obtained i-t curve when 5 mA cm^{-2} was applied in the FOR chamber. (**b**) The corresponding i-t curve in the chemical chamber when 0 V vs $Fc^{+/0}$ was applied.

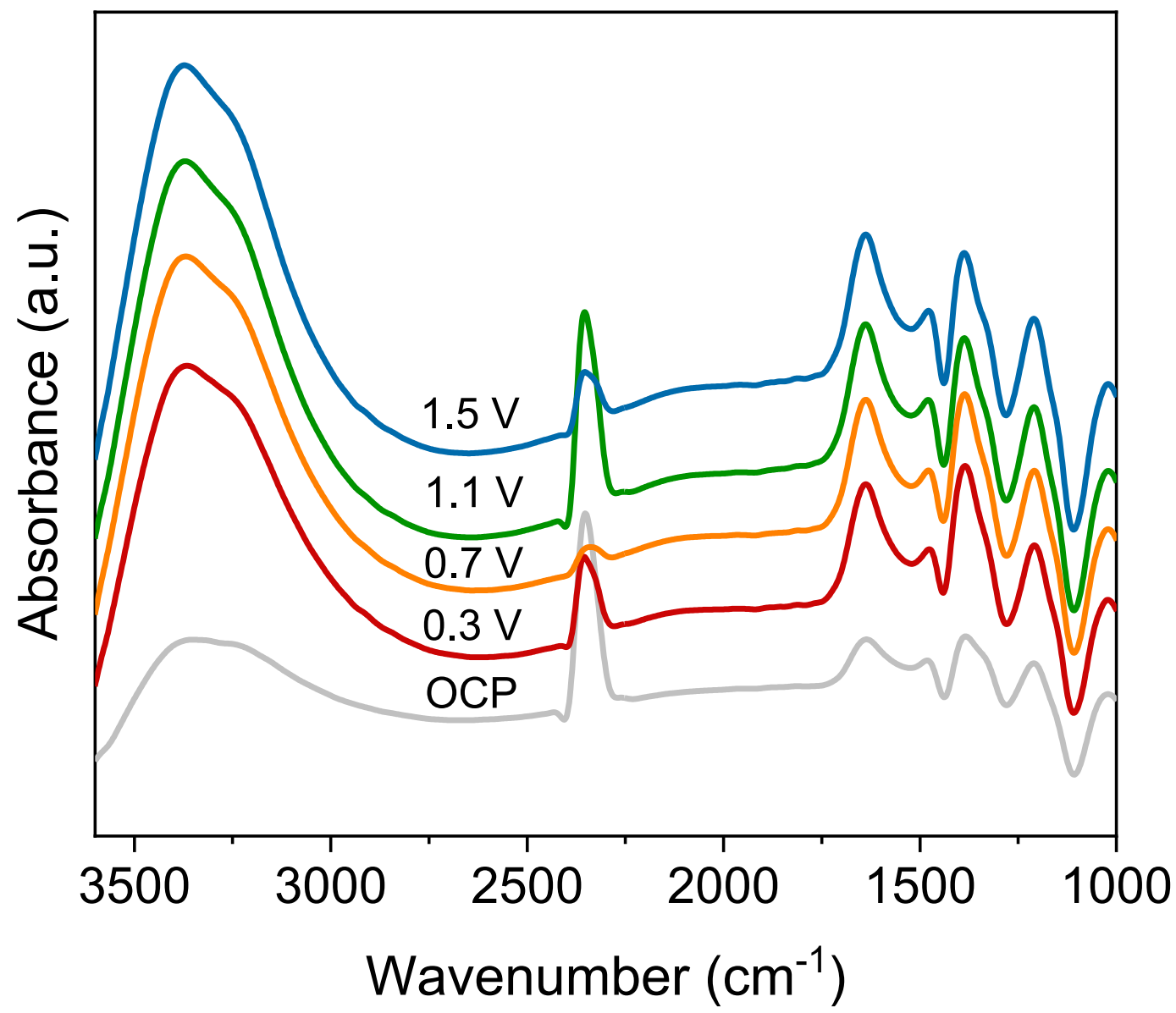

Figure S30. Potential-dependent *in situ* IR spectroscopy recorded on Pd in 1 M KOH without addition of HCHO.

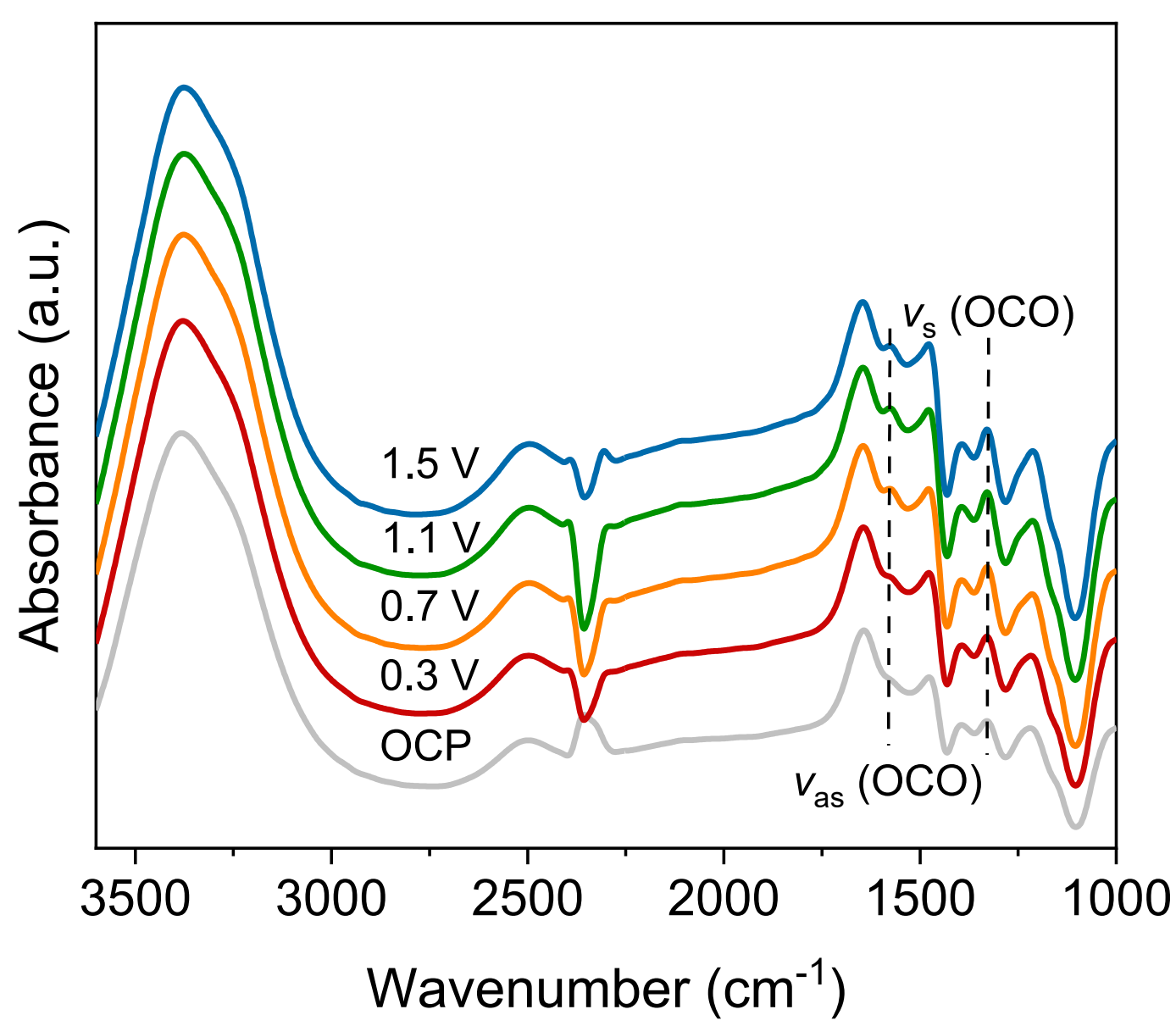

Figure S31. Potential-dependent *in situ* IR spectroscopy recorded on Pd in 1 M KOH with 0.5 M DCDO.

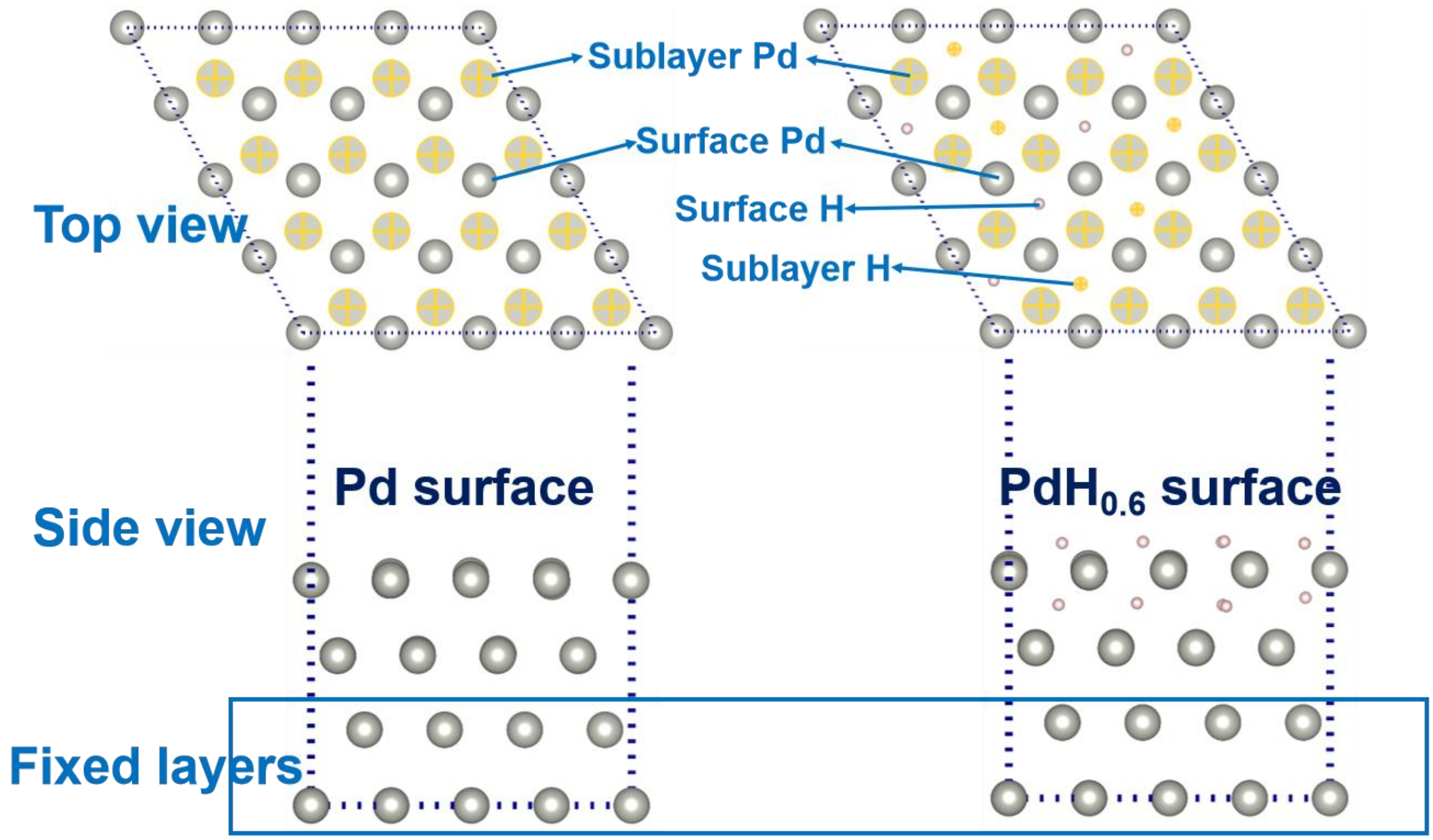

Figure S32. Top and side views of optimized Pd(111) and hydrogen-enriched $PdH_{0.6}$ slab models employed for DFT calculations. The bottom two Pd layers were fixed during structural optimization to represent the bulk Pd, whereas the remaining atoms were fully relaxed. The $PdH_{0.6}$ structure was generated using a combined random hydrogen loading and *ab initio* molecular dynamics (AIMD) sampling strategy followed by full geometry optimization, resulting in hydrogen occupation at both surface and subsurface regions. Gray and pink spheres represent Pd and H atoms, respectively.

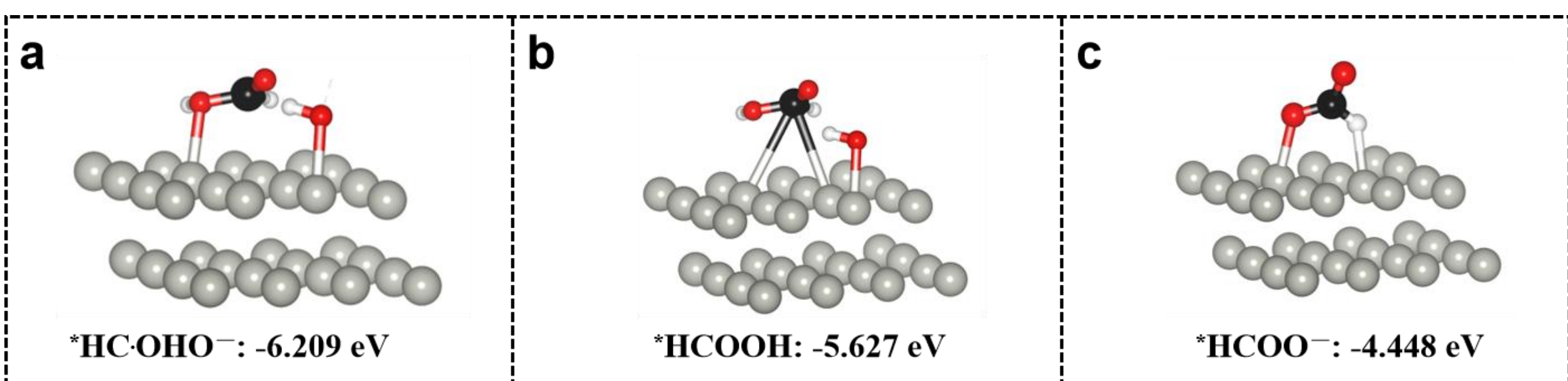

Figure S33. Calculated adsorption energy of (a) *HC·OHO⁻, (b) *HCOOH, and (c) *HCOO⁻ on Pd(111) surfaces.

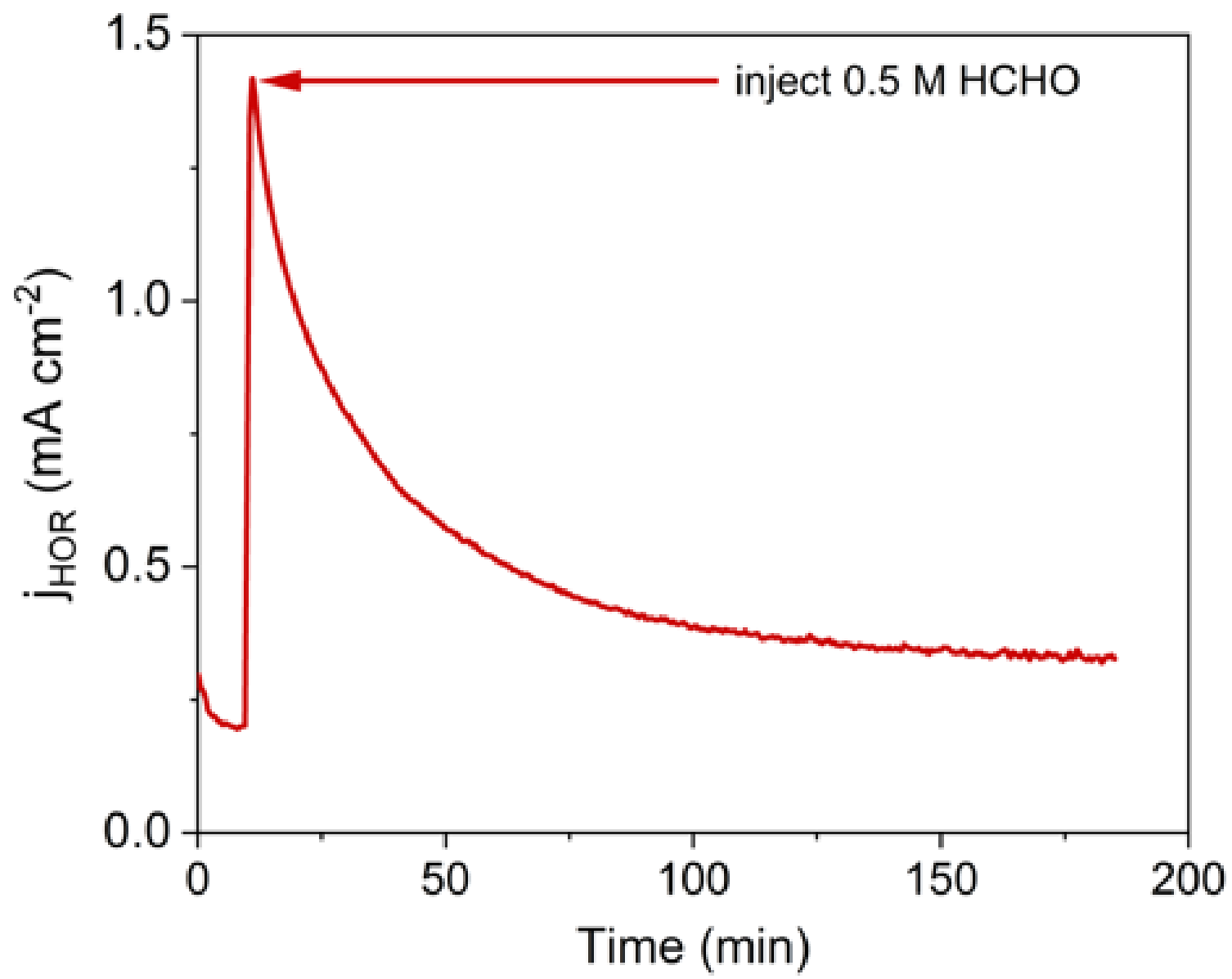

Figure S34. Measured anodic current density in the HOR chamber containing 0.5 M H_2SO_4 upon the addition of 0.5 M HCHO in the FOR chamber without anodic potential applied.

References

1. Kresse, G.; Furthmüller, J. Efficient iterative schemes for ab initio total-energy calculations using a plane-wave basis set. *Phys. Rev. B: Condens. Matter Mater. Phys.* **1996**, *54*, 11169– 11186, DOI: 10.1103/physrevb.54.11169.

2. Blöchl, P. E. Projector augmented-wave method. *Phys. Rev. B: Condens. Matter Mater. Phys.* **1994**, *50*, 17953, DOI: doi.org/10.1103/PhysRevB.50.17953.

3. Perdew, J. P.; Burke, K.; Ernzerhof, M. Generalized Gradient Approximation Made Simple. *Phys. Rev. Lett.* **1996**, *77*, 3865– 3868, DOI: 10.1103/PhysRevLett.77.3865.

4. Henkelman, G.; Uberuaga, B. P.; Jónsson, H. A Climbing Image Nudged Elastic Band Method for Finding Saddle Points and Minimum Energy Paths. *J. Chem. Phys.* **2000**, *113*, 9901– 9904, DOI: 10.1063/1.1329672.
